# Neuromodulatory adaptive combination of correlation-based learning in cerebellum and reward-based learning in basal ganglia for goal-directed behavior control

**DOI:** 10.3389/fncir.2014.00126

**Published:** 2014-10-28

**Authors:** Sakyasingha Dasgupta, Florentin Wörgötter, Poramate Manoonpong

**Affiliations:** ^1^Institute for Physics - Biophysics, George-August-UniversityGöttingen, Germany; ^2^Bernstein Center for Computational Neuroscience, George-August-UniversityGöttingen, Germany; ^3^Center for Biorobotics, Maersk Mc-Kinney Møller Institute, University of Southern DenmarkOdense, Denmark

**Keywords:** decision making, recurrent neural networks, basal ganglia, cerebellum, operant conditioning, classical conditioning, neuromodulation, correlation learning

## Abstract

Goal-directed decision making in biological systems is broadly based on associations between conditional and unconditional stimuli. This can be further classified as classical conditioning (correlation-based learning) and operant conditioning (reward-based learning). A number of computational and experimental studies have well established the role of the basal ganglia in reward-based learning, where as the cerebellum plays an important role in developing specific conditioned responses. Although viewed as distinct learning systems, recent animal experiments point toward their complementary role in behavioral learning, and also show the existence of substantial two-way communication between these two brain structures. Based on this notion of co-operative learning, in this paper we hypothesize that the basal ganglia and cerebellar learning systems work in parallel and interact with each other. We envision that such an interaction is influenced by reward modulated heterosynaptic plasticity (RMHP) rule at the thalamus, guiding the overall goal directed behavior. Using a recurrent neural network actor-critic model of the basal ganglia and a feed-forward correlation-based learning model of the cerebellum, we demonstrate that the RMHP rule can effectively balance the outcomes of the two learning systems. This is tested using simulated environments of increasing complexity with a four-wheeled robot in a foraging task in both static and dynamic configurations. Although modeled with a simplified level of biological abstraction, we clearly demonstrate that such a RMHP induced combinatorial learning mechanism, leads to stabler and faster learning of goal-directed behaviors, in comparison to the individual systems. Thus, in this paper we provide a computational model for adaptive combination of the basal ganglia and cerebellum learning systems by way of neuromodulated plasticity for goal-directed decision making in biological and bio-mimetic organisms.

## 1. Introduction

Associative learning by way of conditioning, forms the main behavioral paradigm that drives goal-directed decision making in biological organisms. Typically, this can be further classified into two classes, namely, classical conditioning (or correlation-based learning) (Pavlov, 1927) and operant conditioning (or reinforcement learning) (Skinner, 1938). In general, classical conditioning is driven by associations between an early occurring conditional stimulus (CS) and a late occurring unconditional stimulus (US), which lead to conditioned responses (CR) or unconditioned responses (UR) in the organism (Clark and Squire, 1998; Freeman and Steinmetz, 2011). The CS here acts as a predictor signal such that, after repeated pairing of the two stimuli, the behavior of the organism is driven by the CR (adaptive reflex action) at the occurrence of the predictive CS, much before the US arrives. The overall behavior is guided on the sole basis of stimulus-response (S-R) associations or correlations, without any explicit feedback in the form of rewards or punishments from the environment. In contrast to such classically conditioned reflexive behavior acquisition, operant conditioning provides an organism with adaptive control over the environment with the help of explicit positive or negative reinforcements (evaluative feedback) given for corresponding actions. Over sufficient time, this enables the organism to respond with good behaviors, while avoiding bad or negative behaviors. As such within the computational learning framework, this is usually termed reinforcement learning (RL) (Sutton and Barto, 1998).

**Figure 1:**
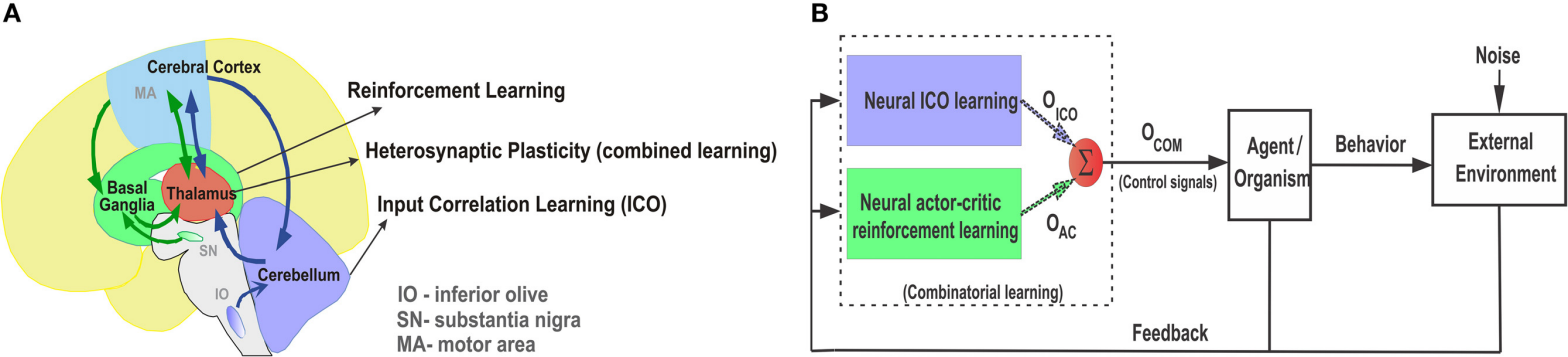
**(A)** Pictorial representation of the anatomical reciprocal connections between the basal ganglia, thalamus, and cerebellum. Green arrows depict the cortico-striatal reward learning circuitry via the thalamus. Blue arrows depict the cortico-cerebellar recurrent loops for classically conditioned reflexive behaviors. Adapted and modified from Doya (2000a). **(B)** Combinatorial learning framework with parallel combination of ICO learning and actor-critic reinforcement learning. Individual learning mechanisms adapt their weights independently and then their final weighted outputs (*O_ico_* and *O_ac_*) are combined into *O_com_* using a reward modulated heterosynaptic plasticity rule (dotted arrows represent plastic synapses). *O_com_* controls the agent behavior (policy) while sensory feedback from the agent is sent back to both the learning mechanisms in parallel.

At a behavioral level, although the two conditioning paradigms of associative learning appear to be distinct from each other, they seem to occur in combination as suggested from several animal behavioral studies (Rescorla and Solomon, 1967; Dayan and Balleine, 2002; Barnard, 2004). Behavioral studies with rabbits (Lovibond, 1983) demonstrate that the strength of operant responses can be influenced by simultaneous presentation of classically conditioned stimuli. This was further elaborated upon in the behavior of fruit flies (Drosophila), where both classical and operant conditioning predictors influence the behavior at the same time and in turn improve the learned responses (Brembs and Heisenberg, 2000). On a neuronal level, this relates to the interaction between the reward modulated action selection at the basal ganglia and the correlation based delay conditioning at the cerebellum. Although the classical notion has been to regard the basal ganglia and the cerebellum to be primarily responsible for motor control, increasing evidence points toward their role in non-motor specific cognitive tasks like goal-directed decision making (Middleton and Strick, 1994; Doya, 1999). Interestingly, recent experimental studies (Neychev et al., 2008; Bostan et al., 2010) show that the the basal ganglia and cerebellum not only form multi-synaptic loops with the cerebral cortex, but, two-way communication between the structures exist via the thalamus Figure 1A) along with substantial disynaptic projections to the cerebellar cortex from the subthalamic nucleus (STN) of the basal ganglia and from the dentate nucleus (cerebellar output stage) to the striatum (basal ganglia input stage) (Hoshi et al., 2005). This suggests that the two structures are not separate performing distinct functional operations (Doya, 2000a), but are linked together forming an integrated functional network. Such integrated behavior is further illustrated in the timing and error prediction studies of Dreher and Grafman (2002) showing that the activation of the cerebellum and basal ganglia are not specific to switching attention, as previously believed, because both these regions were activated during switching between tasks as well as during the simultaneous maintenance of two tasks.

Based on these compelling evidences we formulate the *neural combined learning hypothesis*, which proposes that goal-directed decision making occurs with a parallel adaptive combination (balancing) of the two learning systems (Figure 1B) to guide the final action selection. As evident from experimental studies (Haber and Calzavara, 2009), the thalamus potentially plays a critical role in integrating the neural signals from the two sub-networks while having the ability to modulate behavior through dopaminergic projections from the ventral tagmental area (VTA) (García-Cabezas et al., 2007; Varela, 2014). The motor thalamic (Mthal) relay nuclei, specifically the VA-VL (ventral anterior and ventral lateral) regions receive projections from the basal ganglia (inputs from the globas pallidus) as well as the cerebellum (inputs from the dentate nucleus) (Jones et al., 1985; Percheron et al., 1996). This can be further segregated with the ventral anterior and the anterior region of the ventrolateral nucleus (VLa) receiving major inputs from the globus pallidus internus (GPi), while the posterior region of the ventrolateral nucleus (VLp) receives primary inputs from the cerebellum (Bosch-Bouju et al., 2013). Recent studies using molecular markers were able to distinguish the VA and VL nuclei in rats (Kuramoto et al., 2009), which had hitherto been difficult and were considered as a single overlapping area as the VA-VL complex. Interestingly, despite apparent anatomical segregation of information in the basal ganglia and cerebellar territories, similar ranges of firing rate and movement related activity are observed in the Mthal neurons across all regions (Anderson and Turner, 1991). Furthermore, some experimental studies based on triple labeling techniques found zones of overlapping projections, as well as interdigitating foci of pallidal and cerebellar labels, particularly in border regions of the VLa (Sakai et al., 2000). In light of these evidences, it is plausible that the basal ganglia and cerebellar circuitries not only form an integrated functional network, but their individual outputs are combined together by a subset of the VLa neurons which in turn project to the supplementary and pre-supplementary motor cortical areas (Akkal et al., 2007) responsible for goal-directed movements. We envision that such a combined learning mechanism may be driven by reward modulated heterosynaptic plasticity (neuromodulation by way of dopaminergic projections) at the thalamus.

In this study, input correlation learning (ICO)in the form of a differential Hebbian learner (Porr and Wörgötter, 2006), was implemented as an example of delay conditioning in the cerebellum, while a reservoir network (Jaeger and Haas, 2004) based continuous actor-critic learner (Doya, 2000b) was implemented as an example of reward based conditioning in the basal ganglia. Taking advantage of the individual learning mechanisms, the combined framework can learn the appropriate goal-directed control policy for an agent^1^ in a fast and robust manner outperforming the singular implementation of the individual components.

Although there have been a number of studies which have applied the two different conditioning concepts for studying self-organizing behavior in artificial agents and robots, they have mostly been applied separately to generate specific goal-directed behaviors (Morimoto and Doya, 2001; Verschure and Mintz, 2001; Hofstoetter et al., 2002; Prescott et al., 2006; Manoonpong et al., 2007; Soltoggio et al., 2013). In our previous work (Manoonpong et al., 2013) we motivated a combined approach of the two learning concepts on a purely algorithmic level without any adaptive combination between the two. To the best of our knowledge, in this paper we present for the first time a biologically plausible approach to model an adaptive combination of the cerebellar and basal ganglia learning systems, where they indirectly interact through sensory feedback. In this manner they work as a single functional unit to guide the behavior of artificial agents. We test our neural combined learning hypothesis within the framework of goal-directed decision making using a simulated wheeled robot situated in environments of increasing complexity designed as part of static and dynamic foraging tasks (Sul et al., 2011). Our results clearly show that the proposed mechanism enables the artificial agent to successfully learn the task in the different environments with changing levels of interaction between the two learning systems. Although we take a simplified approach of simulated robot based goal-directed learning, we believe our model covers a reasonable level of biological abstraction that can help us understand better, the closed-loop interactions between these two neural subsystems as evident from experimental studies and also provide a computational model of such combined learning behavior which has hitherto been missing.

We now give a brief introduction to the neural substrates of the cerebellum and the basal ganglia with regards to classical and operant conditioning. Using a broad high-level view of the anatomical connections of these two brain structures, we motivate how goal-directed behavior is influenced by the respective structures and their associated neuronal connections. The individual computational models with implementation details of the two interacting learning systems are then presented in the Materials and Methods Section followed by results and discussion.

### 1.1. Classical conditioning in the cerebellum

**Figure 2:**
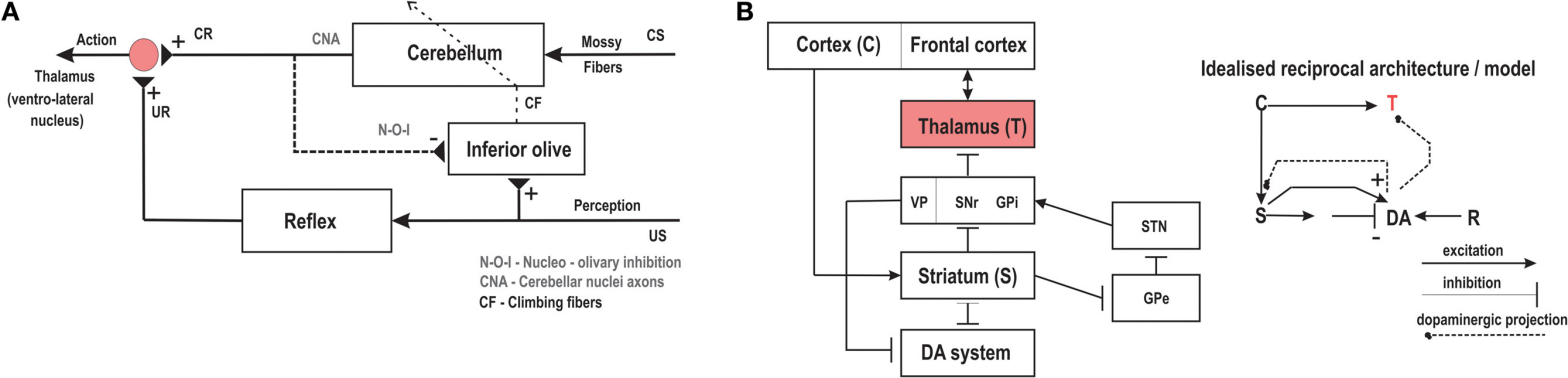
**(A)** Schema of the cerebellar controller with the reflexive pathways and anatomical projections leading the acquisition of reflexive behaviors. CS, conditioned stimulus; US, unconditioned stimuli; CR, conditioned response; UR, unconditioned response. **(B)** (right) Schematic representation of the neural architecture of the basal ganglia circuitry showing the layout of the various internal connections. (left) Shows the simplified circuit diagram with the main components as modeled in this paper using the reservoir actor-critic framework. C, Cortex; S, striatum; DA, dopamine system; R, reward; T, thalamus. Adapted and modified from Wörgötter and Porr (2005).

The role of the Cerebellum and its associated circuitry in the acquisition and retention of anticipatory responses (sensory predictions) with Pavlovian delay conditioning has been well established (Christian and Thompson, 2003; Thompson and Steinmetz, 2009). Although most of the classical conditioning studies are primarily based on eye-blink conditioning (Yeo and Hesslow, 1998), recent experimental studies have established the essential role of the cerebellum in learning and memory of goal-directed behavioral responses (Burguiere et al., 2010). In Figure 2A a highly simplified control structure of the major cerebellar pathways and their relative function is indicated. The Inferior Olive relays the US signal to the cerebellar cortex through the climbing fibers and then induces plasticity at the synaptic junctions of the mossy fibers carrying the CS information (Herreros and Verschure, 2013). Repeated CS-US pairings gradually lead (through synaptic consolidation) to the acquisition of the CR with a drop in the firing activity of the Purkinje cells (output from the cerebellar cortex). The cerebral cortex projects to the lateral cerebellum via pontine nuclei relays (Allen and Tsukahara, 1974; Lisberger and Thach, 2013; Proville et al., 2014) which in turn have projections back to the cerebral cortex through relays in the thalamus (ventro-lateral nucleus), thus projecting the conditioned responses from the cerebellum to the motor cortical areas (Stepniewska et al., 1994; Sakai et al., 2000). In essence, the cerebellar action modulates or controls the motor activity of the animal which produces changes in its goal oriented behavior. The goal oriented behaviors can typically involve both attraction toward or avoidance of specific actions (generally referred to as adaptive reflexes) involving both sensory predictions and motor control, toward which the cerebellum makes a major contribution. It is also important to note that although numerous experimental and computational studies demonstrate the function of the Cerebellum in classical conditioning or correlation learning (Kim and Thompson, 1997; Woodruff-Pak and Disterhoft, 2008), a possible role of the Cerebellum toward supervised learning (SL) has also been widely suggested (Doya, 1999; Kawato et al., 2011). Typically within the paradigm of SL a training or instructive signal acts as a reference toward which the output of a network (movements) is compared, such that the error generated acts as the driver signal to induce plasticity within the network in order to find the correct mapping between the sensory input stimuli and the desired outputs (Knudsen, 1994). Using the classical conditioning paradigm, it has been suggested that the instructive signal that supervises the learning is the input activity associated with the US. As such, the SL model of the cerebellum considers that the climbing fibers from the inferior olive provide the error signal (instructive activity) for the Purkinje cells. Coincident inputs from the inferior olive and the granule cells lead to plasticity at the granule-to-Purkinje synapses (Doya, 2000a). Although there have been experimental studies to validate the SL description of the cerebellum (Kitazawa et al., 1998), it has been largely directed toward considering the cerebellum as an internal model of the body and the environment (Kawato, 1999). Furthermore, Krupa et al. (1993) observed that even when the red nucleus (relay between motor cortex and cerebellum) was inactivated learning proceeded with no CR being expressed. Thus, this demonstrates that no error signal based on the behavior was needed for learning to occur. Instead, the powerful climbing fiber activity evoked by the US, acting as a template, could cause the connection strengths of sensory inputs that are consistently correlated with it to increase. Subsequently, after sufficient repetition, the activity of these sensory inputs alone would drive the UR pathway. As such, in this work we directly consider correlation learning as the basis of classical conditioning in the cerebellum without taking into consideration SL mechanisms and do not explicitly consider the US relay from the inferior olive as an error signal.

### 1.2. Reward learning in the basal ganglia

In contrast to the role of the cerebellum in classical conditioning, the basal ganglia and its associated circuitry possess the necessary anatomical features (neural substrates) required for a reward-based learning mechanism (Schultz and Dickinson, 2000). In Figure 2B we depict the main anatomical connections of the cortical basal ganglia circuitry. It is comprised of the striatum (consisting of most of the caudate and the putamen, and of the nucleus accumbens), the internal (medial) and external (lateral) segments of the globus pallidus (GPi and GPe respectively), the subthalamic nucleus (STN), the ventral tegmental area (VTA) and the substantia nigra pars compacta (SNc) and pars reticulata (SNr). The input stage of the basal ganglia is the striatum connected via direct cortical projections. Previous studies have not only recognized the striatum as a critical structure in the learning of stimulus-response behaviors, but also established it as the major location which projects to as well as receives efferent connections from (via direct and indirect multi-synaptic pathways) the dopaminergic system (Joel and Weiner, 2000; Kreitzer and Malenka, 2008). The processing of rewarding stimuli is primarily modulated by the dopamine neurons (DA system in Figure 2B) of the VTA and SNc with numerous experimental studies (Schultz and Dickinson, 2000) demonstrating, that changes in dopamine neurons encode the prediction error in appetitive learning scenarios, and associative learning in general (Puig and Mille, 2012). Figure 2B—right shows the idealized reciprocal architecture of the striatal and dopaminergic circuitry. Here sensory stimuli arrive as input from the cortex to the striatal network. Excitatory as well as inhibitory synapses project from the striatum to the DA system which in turn uses the changes in the activity of DA neurons to modulate the activity in the striatum. Such DA activity also acts as the neuromodulatory signal to the thalamus which receives indirect connections from the striatum, via the GPi, SNr and VTA (Varela, 2014). Computational modeling of such dopamine modulated reward learning behavior is particularly well reflected by the Temporal Difference (TD) algorithm (Sutton, 1988; Suri and Schultz, 2001), as well as in the action selection based computational models of the basal ganglia (Gurney et al., 2001; Humphries et al., 2006). In the context of basal ganglia modeling, Actor-Critic models (explained further in the next section) of TD learning (Houk et al., 1995; Joel et al., 2002) have been extensively used. They create a functional separation between two sub-networks of the critic (modeling striatal and dopaminergic activity) and the actor (modeling striatal to motor thalamus projections). The TD learning rule uses the prediction error (TD error) between two subsequent predictions of the net weighted sum of future rewards based on current input and actions, to modulate critic weights via long-term synaptic plasticity. The same prediction error signal (dopaminergic projections) is also used to modulate the synaptic weights at the actor; output from which controls the the actions taken by the agent. Typically, here the mechanism of action selection, can be regarded as the neuromodulation process occurring at the striatum, which then reaches the motor thalamic regions via projections from the output stages of the basal ganglia, namely GPi/GPe and SNr (Gurney et al., 2001; Houk et al., 2007) (Figure 2B).

## 2. Materials and methods

### 2.1. Combinatorial learning with reward modulated heterosynaptic plasticity

**Figure 3:**
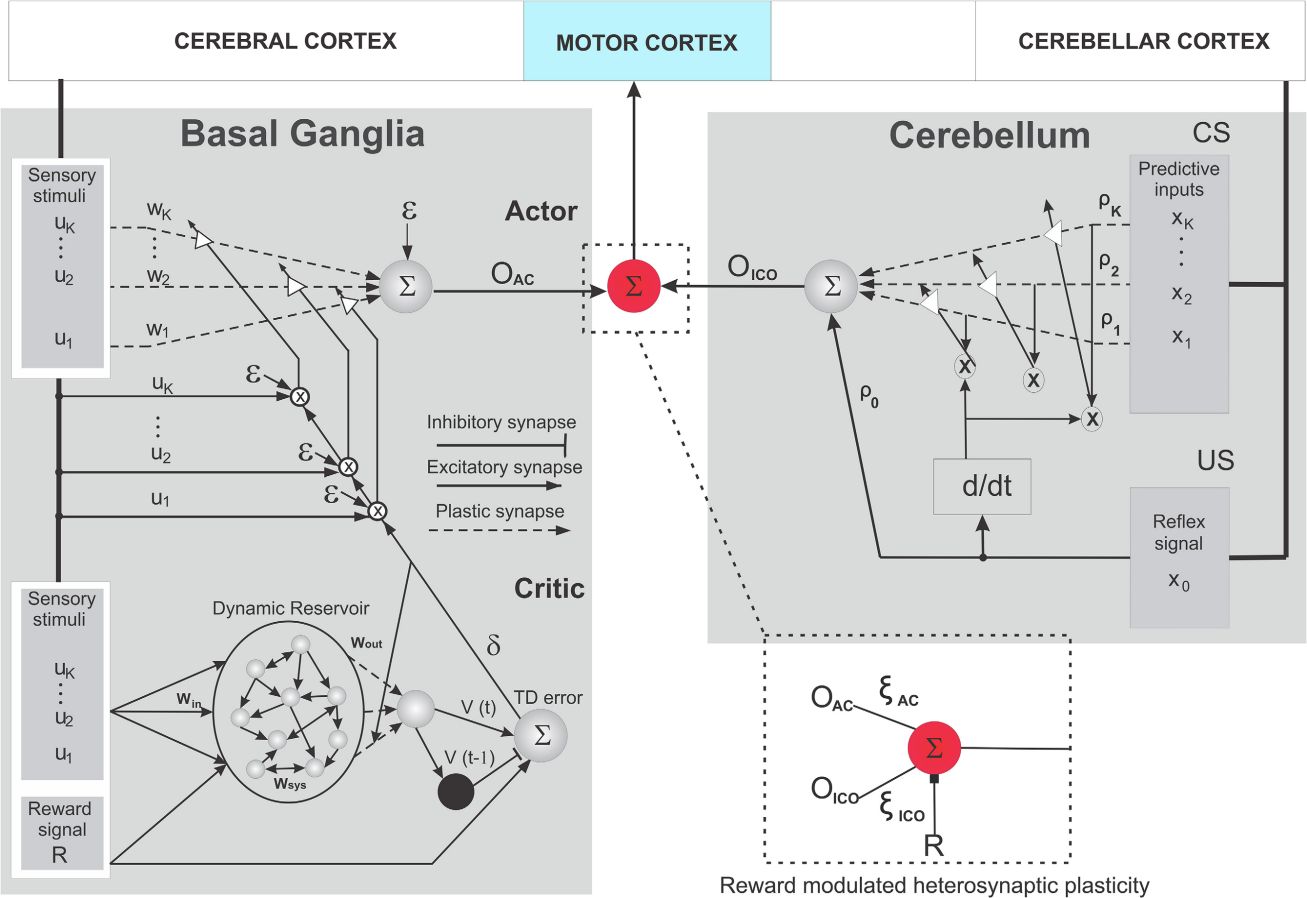
**Schematic wiring diagram of the combined learning neural circuit**. It consists of the reservoir actor-critic RL based on TD learning **(left)** and the input correlation learning (ICO) **(right)** models. The critic here is reminiscent of the cortico striatal connections modulated by dopaminergic neural activity (TD error). The actor represents the neuromodulation process at the striatum, which reaches the motor thalamus by projections from GPi/GPe and SNr. The ICO learning system is constructed in a manner similar to Figure 2A, with the inferior olive being represented by the differential Hebbian (d/dt) system that uses the US reflex signal to modulate the synaptic connections in the cerebellum. Explicit nucleo-olivary inhibitory connections were not modeled here. The red circle represents the communication junction which act as the integrator of the outputs from the two networks, being directly modulated by the reward signal R to control the overall action of the agent. (further details in text).

According to the neural combined learning hypothesis for successful goal-directed decision making, the underlying neural machinery of animals combines basal ganglia and cerebellar learning systems output, induced with a reward modulated balancing (neuromodulation) between the two, at the thalamus to achieve net sensory-motor adaptation. Thus, here we develop a system for the parallel combination of the input correlation-based learner (ICO) and the reward-based learner (actor-critic) as depicted in Figure 1B. The system works as a dual learner where the individual learning mechanisms run in parallel to guide the behavior of the agent. Both systems adapt their synaptic weights independently (as per their local synaptic modification rules) while receiving the same sensory feedback from the agent (environmental stimuli) in parallel. The final action that drives the agent is calculated as a weighted sum (Figure 3 red circle) of the individual learning components. This can be described as follows:

(1) $$o_{com}(t)=\xi_{ico}o_{ico}(t)+\xi_{ac}o_{ac}(t)$$

where, *o_ico_*(*t*) and *o_ac_*(*t*) are the *t* time step outputs of the input correlation-based learner and the actor-critic reinforcement learner, respectively. *o_com_*(*t*) represents the *t* time step combined action. The key parameters here that govern the learning behavior are the synaptic weights of the output neuron projection from the individual components (ξ*_ico_* and ξ*_ac_*). These govern the degree of influence of the two learning systems, on the net action of the agent. Previously, a simple and straight forward approach was undertaken in Manoonpong et al. (2013), where an equal contribution (ξ_*ico*_ = ξ_*ac*_ = 0.5) of ICO and actor-critic RL for controlling an agent was considered. Although this can lead to successful solutions in certain goal-directed problems, it is sub-optimal due to the lack of any adaptive balancing mechanism. Intuitively for associative learning problems with immediate rewards the ICO system learns quickly as compared to distal reward based goal-directed problems where, the ICO learner can provide guidance to the actor-critic learner. In particular depending on the type of problem, the right balance between the two learners needs to be achieved in an adaptive manner.

While there is evidence on the direct communication (Bostan et al., 2010) or combination of the subcortical loops from the cerebellum and the basal ganglia (Houk et al., 2007), a computational mechanism underlying this combination has not been presented, so far. Here we propose for the first time, an adaptive combination mechanism of the two components, modeled in the form of a reward modulated heterosynaptic plasticity (RMHP) rule, which learns the individual synaptic weights (ξ*_ico_* and ξ*_ac_*) for the projections from these two components. It is plausible that such a combination occurs at the VA-VL region of the motor thalamic nuclei which has both pallido-thalamic (basal ganglia) and cerebello-thalamic projections (Sakai et al., 2000). Furthermore, a few previous experimental studies (Desiraju and Purpura, 1969; Allen and Tsukahara, 1974) suggested that the individual neurons of the VL (nearly 20%) integrate signals from the basal ganglia and the cerebellum along with some weak cerebral inputs^2^. Based on biological evidence of dopaminergic projections at the thalamus from the basal ganglia circuit (García-Cabezas et al., 2007; Varela, 2014) as well as cerebellar projections to the thalamic ventro-latral nucleus (Bosch-Bouju et al., 2013) (see Figures 42–47 in Lisberger and Thach, 2013) we consider here that such dopaminergic projections act as the neuromodulatory signal and triggers the heterosynaptic plasticity (Ishikawa et al., 2013). A large number of such heterosynaptic plasticity mechanisms contribute toward a variety of neural processes involving associative learning and development of neural circuits in general (Bailey et al., 2000; Chistiakova and Volgushev, 2009). Although there is currently no direct experimental evidence of heterosynaptic plasticity at thalamic nuclei, it is highly plausible that such interactions could occur on synaptic afferents as observed in the amygdala and the hippocampus (Vitureira et al., 2012). Here, we use the instantaneous reward signal as the modulatory input in order to induce heterosynaptic changes at the thalamic junction. Similar approach have also been used in some previous theoretical models of reward modulated plasticity (Legenstein et al., 2008; Hoerzer et al., 2012). Although the dopaminergic projections from the VTA to the Mthal are primarily believed to encode a reward prediction error (RPE) signal (Schultz and Dickinson, 2000), there exists considerable diversity in the VTA neuron types with a subset of these dopaminergic neurons directly responding to rewards (Cohen et al., 2012). Similar variability has also been observed in the single DA neuron recordings from memory guided sacadic tasks performed with primates (Takikawa et al., 2004). This suggests that although most dopaminergic neurons respond to a reward predicting conditional simuli, some may not strictly follow the canonical RPE coding (Cohen et al., 2012). It is important to note that, within this model, it is equally possible to use the reward prediction error (TD error, Equation 12) and still learn the synaptic weights of the two components in a stable manner, however with a negligibly slower weight convergence due to continuous weight changes (see Supplementary Figure 1).

Based on this RMHP plasticity rule the ICO and actor-critic RL weights are learned at each time step as follows:

(2) $$\Delta \xi_{ico}(t)\;=\eta r(t)[o_{ico}(t)-{\bar{o}}_{ico}(t)]o_{ac}(t),$$

(3) $$\Delta \xi_{ac}(t)\;=\eta r(t)[o_{ac}(t)-{\bar{o}}_{ac}(t)]o_{ico}(t).$$

Here *r*(*t*) is the current time step reward signal received by the agent, while *o**_ico_*(*t*) and *o*_*ac*_(*t*) denote the low-pass filtered version of the output from the ICO learner and the actor-critic learner, respectively. They are calculated as:

(4) $$\begin{array}{l} {\bar{o}}_{ico}(t)=0.9{\bar{o}}_{ico}(t-1)+0.1o_{ico}(t),\\ \;{\bar{o}}_{ac}(t)=0.9{\bar{o}}_{ac}(t-1)+0.1o_{ac}(t). \end{array}$$

The plasticity model used here is based on the assumption that the net policy performance (agent's behavior) is influenced by a single global neuromodulatory signal. This relates to the dopaminergic projections to the ventra-lateral nucleus in the thalamus as well as connections from the amygdala which can carry reward related signals that influence over all action selection. The RMHP learning rule correlates three factors: (1) the reward signal, (2) the deviations of the ICO and actor-critic learner outputs from their mean values, and (3) the actual ICO and actor-critic outputs. The correlations are used to adjust their respective synaptic weights (ξ*_ico_* and ξ*_ac_*). Intuitively here the heterosynaptic plasticity rule can be also viewed as a homeostatic mechanism (Vitureira et al., 2012). Such that, the equation 2 tells the system to increase the ICO learners weights (ξ*_ico_*) when the ICO output is coincident with the positive reward, while the third factor (*o_ac_*) tells the system to increase ξ*_ico_* more (or less) when the actor-critic learner weights (ξ*_ac_*) are large (or small), and vice versa for Equation 3. This ensures that overall the ratio of weight change of the two learning components occurs at largely the same rate. Additionally in order to prevent uncontrolled divergence in the learned weights, homeostatic synaptic normalization is carried out specifically as follows:

(5) $$\begin{array}{l} \xi_{ico}(t)=\frac{\xi_{ico}(t)}{\xi_{ico}(t)+\xi_{ac}(t)},\\ \;\xi_{ac}(t)=\frac{\xi_{ac}(t)}{\xi_{ico}(t)+\xi_{ac}(t)}. \end{array}$$

This ensures that the synaptic weights always add up to one and 0 < ξ_*ico*_, ξ*_ac_* < 1. In general this plasticity rule occurs on a very slow time scale which is governed by the learning rate parameter η. Typically convergence and stabilization of weights are achieved by setting η much smaller compared to the learning rate of the two individual learning systems (ICO and actor-critic). To get a more detailed view of the implementation of the adaptive combinatorial learning mechanism, interested readers should refer to algorithm 2 in the Supplementary Material.

### 2.2. Input correlation model of cerebellar learning

In order to model classical conditioning of adaptive motor reflexes^3^ in the cerebellum, we use a model-free, correlation based, predictive control learning rule called input correlation learning (ICO) (Porr and Wörgötter, 2006). ICO learning provides a fast and stable mechanism in order to acquire and generate sensory predictions for adaptive responses based solely on the correlations between incoming stimuli. The ICO learning rule (Figure 3 Right) takes the form of an unsupervised synaptic modification mechanism using the cross-correlation between the incoming predictive input stimuli (predictive here means that the signals occur early) and a single reflex signal (late occurring). As depicted in Figure 3 right, cortical perceptual input in the form of predictive signals (CS) represents the mossy fiber projections to the cerebellum microcircuit, while the Climbing fiber projections from the inferior olive that modulates the synaptic weights in the deep cerebellar nucleus are depicted in a simplified form with the differential region (*d*/*dt*).

The goal of the ICO mechanism is to behave as a forward model system (Porr and Wörgötter, 2006) that uses the sensory CS to predict the occurrence of the innate reflex signal (external predefined feedback signaling unwanted scenarios), thus letting the agent to react in an anticipatory manner to avoid the basic reflex altogether. Based on a differential Hebbian learning rule (Kolodziejski et al., 2008) the synaptic weights in the ICO scheme are modified using heterosynaptic interactions of the incoming inputs, depending on their order of occurrence. In general, the plastic synapses of the predictive inputs get strengthened if they precede the reflex signal and are weakened if their order of occurrence is reversed. As a result, the ICO learning rule drives the behavior depending on the timing of correlated neural signals. This can be formally represented as,

(6) $$o_{ico}(t)=\rho_{0}x_{0}(t)+\sum_{j\;=\;1}^{K}\rho_{j}(t)x_{j}(t).$$

Here, *o_ico_* represents the output neuron activation of the ICO system driven by the superposition of the plastic K-dimensional predictive inputs *x_j_*(*t*) = *x*_1_(*t*), *x*_2_(*t*), …, *x_K_*(*t*)^4^ (differentially modified) and the fixed innate reflex signal *x*_0_(*t*). The synaptic strength of the reflex signal is represented by ρ_0_ and is fixed to the constant value of 1.0 in order to signal innate response to the agent. Using the cross-correlations between the input signals, our differential Hebbian learning rule modifies synaptic connections as follows:

(7) $$\Delta \rho_{j}(t)=\mu x_{j}(t)\frac{d}{dt}x_{0}(t).$$

Here, μ defines the learning rate and is typically set to a small value to allow slow growth of synaptic weights with convergence occurring once the reflex signal *x_o_* = 0 (Porr and Wörgötter, 2006). Thus, ICO learning allows the agent to predict the primary reflex and successfully generate early, adaptive actions. However, no explicit feedback of goodness of behavior is provided to the agent and thus only an anticipatory response can be learned without the explicit notion of how well the action allows reaching a desired (rewarding) goal location. As depicted in Figure 3, the output from the ICO learner is directly fed into the RMHP unit envisioned to be part of the ventro-lateral thalamic nucleus (Akkal et al., 2007; Bosch-Bouju et al., 2013).

### 2.3. Actor-critic reservoir model of basal-ganglia learning

TD learning (Sutton, 1988; Suri and Schultz, 2001), in the framework of actor-critic reinforcement learning (Joel et al., 2002; Wörgötter and Porr, 2005), is the most established computational model of the basal ganglia. As explained in the previous section, the TD learning technique is particularly well suited for replicating or understanding how reward related information is formed and transferred by the mid-brain dopaminergic activity.

The model consists of two sub-networks, namely, the adaptive critic (Figure 3 left, bottom) and the actor (Figure 3 left, above). The critic is adaptive in the sense that it learns to predict the weighted sum of future rewards taking into account the current incoming sensory stimuli and the actions (behaviors) performed by the agent within a particular environment. The difference between the predicted “value” of sum of future rewards and the actual measure acts as the temporal difference (TD) prediction error signal that provides an evaluative feedback (or reinforcement signal) to drive the actor. Eventually the actor learns to perform the proper set of actions (policy^5^) that maximize the weighted sum of future rewards as computed by the critic. The evaluative feedback (TD error signal) in general acts as a measure of goodness of behavior that, overtime, lets the agent learn to anticipate reinforcing events. Within this computational framework, the TD prediction error signal and learning at the critic are analogous to the dopaminergic (DA) activity and the DA dependent long term synaptic plasticity in the striatum (Figure 2B), while the remaining parts of striatal circuitry can be envisioned as the actor which uses the TD modulated activity to generate actions, which drives the agent's behavior.

Inspired by the reservoir computing framework (Maass et al., 2002; Jaeger and Haas, 2004), here we use a chaotic random recurrent neural network (RNN) (Sussillo and Abbott, 2009; Rajan et al., 2010) as the adaptive critic (cortico-striatal circuitry and the DA system) connected to a feed-forward neural network, serving the purpose of the part of striatum that performs action selection (Gurney et al., 2001) and then relays it to the motor thalamus via projections from the globus pallidus and substantia nigra. This provides an effective framework to model a continuous actor-critic reinforcement learning scheme, which is particularly suited for goal-directed learning in continuous state-action problems, while at the same time maintaining a reasonable level of biological abstraction (Fremaux et al., 2013). Here, the reservoir network can be envisioned as analogous to the cortex and its inherent recurrent connectivity structure, and the readout neurons serving as the striatum, with plastic projections from the recurrent layer, as the modifiable cortico-striatal connections (Hinaut and Dominey, 2013). The reservoir network is constructed as a generic network model of *N* recurrently connected neurons with high sparsity (refer to Supplementary Material for details) and fixed synaptic connectivity. The connections within the recurrent layer are drawn randomly in order to generate a sparsely connected network of inhibitory and excitatory synapses. A subset of the reservoir neurons receive input connections (fixed synaptic strengths) as external driving signals and has an additional output layer of neurons that learns to produce a desired response based on synaptic modification of weights from the reservoir to output neurons. The input connections along with the large recurrently connected reservoir network represents the main cortical microcircuit-to-striatum connections, while the output layer neural activity can be envisioned as striatal neuronal responses. In this case, the reservoir critic provides an input (sensory stimuli) driven dynamic network with a large repertoire of signals that is used to predict the value function *v* (average sum of future rewards). *v*(*t*) approximates the accumulated sum of the future rewards *r*(*t*) with a given discount factor γ (0 ≤ γ < 1)^6^ as follows:

(8) $$v(t)=\sum_{i\;=\;1}^{\infty }\gamma^{i\;-\;1}r(t+i).$$

In our model, the membrane potential at the soma (at time *t*) of the reservoir neurons, resulting from the incoming excitatory and inhibitory synaptic inputs, is given by the *N* dimensional vector of neuron state activation's, **x**(*t*) = *x*_1_(*t*), *x*_2_(*t*), …, *x_N_*(*t*). The input to the reservoir network, consisting of the agent's states (sensory input stimuli from the cerebral cortex), is represented by the *K* dimensional vector **u**(*t*) = *u*_1_(*t*), *u*_2_(*t*), …, *u_K_*(*t*). The recurrent neural activity within the dynamic reservoir varies as a function of its previous state activation and the current driving input stimuli. The recurrent network dynamics is given by,

(9) $$\tau \dot{x}(t)\;=-x(t)+gW_{sys}z(t)+W_{in}u(t)+b,$$

(10) $$\hat{v}(t)\;=\text{tanh}(W_{out}z(t)),$$

(11) $$z_{i}(t)\;=\text{ tanh}(\alpha x_{i}(t)+\beta ).$$

The parameters **W**_*in*_ and **W***_sys_* denote the input to reservoir synaptic weights and the recurrent connection weights within the reservoir, respectively. The parameter *g* (Sompolinsky et al., 1988) acts as the scaling factor for the recurrent connection weights allowing different dynamic regimes from stable to chaotic being present in the reservoir. Similar to Sussillo and Abbott (2009) we select *g* such that the network exhibits chaotic dynamics as spontaneous behavior before learning and maintains stable dynamics after learning, with the help of feedback connections and neuronal activation homeostasis via intrinsic plasticity (Triesch, 2005; Dasgupta et al., 2013a). The RNN does not explicitly model action potentials, but describes neuronal firing rates, where in, the continuous variable *z_i_* is the instantaneous firing rate of the reservoir neurons and is calculated as a non-linear saturating function of the state activation *x_i_* (Equation 11). The output layer consists of a single neuron whose firing rate $\hat{v}$(*t*) is calculated at time *t* based on equation 10, as a non-linear transformation of the weighted projections of the internal reservoir neuron firing rates **z**(*t*). Here the parameter **W**_*out*_ denotes the *N* × *K* dimensional reservoir to output connection synaptic weights. Each unit in the network also receives a constant bias signal *b_i_*, represented in equation 9 as the *N* dimensional vector **b**. The overall time scale of the RNN and the leak rates of individual reservoir neurons are controlled by the parameter τ.

Based on the TD learning principle, the primary goal of the reservoir critic is to predict *v*(*t*) such that the TD error δ is minimized over time. At each time point *t*, δ is computed from the current ($\hat{v}$(*t*)) and previous ($\hat{v}$(*t* − 1)) value function predictions (reservoir output), and the current reward signal *r*(*t*), as follows:

(12) $$\delta (t)=r(t)+\gamma \hat{v}(t)-\hat{v}(t-1).$$

The output weights **W**_*out*_ are calculated using the recursive least squares (RLS) algorithm (Haykin, 2002) at each time step, while the sensory stimuli **u**(*t*) are being fed into the reservoir. **W**_*out*_ are calculated such that the overall TD-error (δ) is minimized. We implement the online RLS algorithm using a fixed forgetting factor (λ*_RLS_* < 1) as given in Algorithm 1.

**Algorithm 1:** **Online RLS algorithm for learning reservoir to output neuron weights**.

*Initialize*: **W***_out_* = 0, exponential forgetting factor (λ*_RLS_*) is set to a value less than 1 (we use 0.85) and the auto-correlation matrix ρ is initialized as ρ(0) = **I**/β, where **I** is unit matrix and β is a small constant.
*Repeat*: At time step *t*
Step 1: For each input signal **u**(*t*), the reservoir neural firing rate vector **z**(*t*) and network output $\hat{v}$(*t*) are calculated using equation 11 and equation 10.
Step 2: Online error *e*(*t*) calculated as:
*e*(*t*) ← δ (*t*)
Step 3: Gain vector **K**(*t*) is updated as:
$K(t)\leftarrow \frac{\rho (t-1)z(t)}{\lambda_{RLS}+z^{T}(t)\rho (t-1)z(t)}$
Step 4: Update the auto-correlation matrix ρ (*t*)
$\rho (t)\leftarrow \frac{1}{\lambda_{RLS}}\left[\rho (t-1)-K(t)z^{T}(t)\rho (t-1)\right]$
Step 5: Update the instantaneous output weights **W**_*out*_(*t*)
**W**_*out*_(*t*) ← **W**_*out*_(*t* − 1) + *K*(*t*)*e*(*t*)
Step 6: *t* ← *t* + 1
*Until*: The maximum number of time steps is reached.

**Figure 4:**
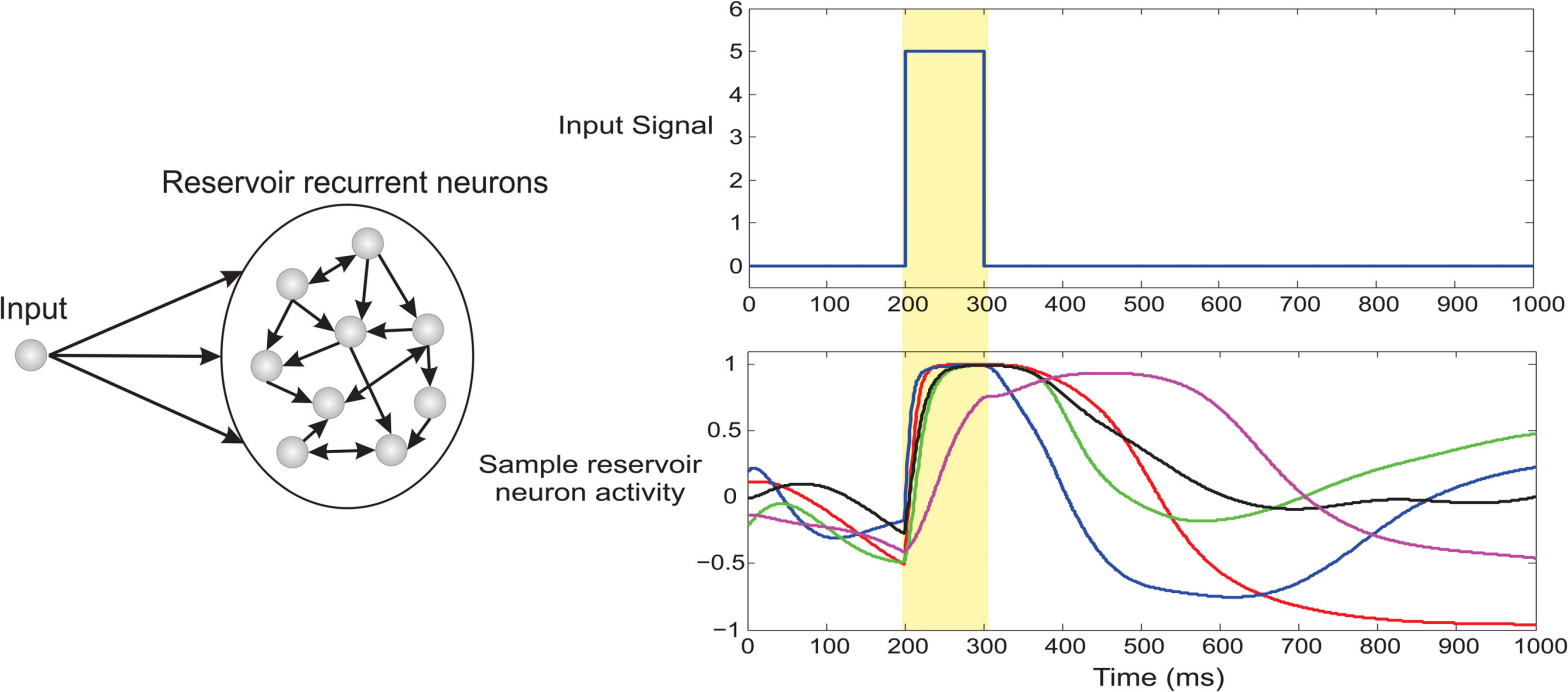
**Fading temporal memory in recurrent neurons of dynamic reservoir**. The recurrent network (100 neurons) was driven by a brief 100 ms pulse and a fixed auxiliary input of magnitude 0.3 (not shown here). Spontaneous dynamics then unfolds in the system based on Equation 9. The lower right panel plots the activity of 5 randomly selected recurrent neurons. It can be clearly observed that the driving input signal clamps the activity of the network at 200 ms however different neurons decay with varying timescale. As a result the network exhibits considerable fading memory of the brief incoming input stimuli.

As proposed in Triesch (2005) and Dasgupta et al. (2013a) we implement a generic intrinsic plasticity mechanism based on the Weibull distribution for unsupervised adaptation of the reservoir neuron non-linearity using a stochastic decent algorithm to adapt the scale α and shape parameters β of the saturating function in Equation 11. This allows the reservoir to homoeostatically maintain a stable firing rate while at the same time it drives the neuron activities to a non-chaotic regime. It is also important to note that one of the primary assumptions of the basic TD learning rule is a Markovian one, which considers future sensory cues and rewards depending only on the current sensory cue without any memory component. The use of a reservoir critic (due to the inherent fading temporal memory of recurrent networks Lazar et al., 2007) breaks this assumption. As a result, such design principle extends our model to problems with short term dependence of immediate sensory stimuli on the preceding history of stimuli and reward (see Figure 4 for a simulated example of local temporal memory in reservoir neurons).

The actor (Figure 3 left above) is designed as a single stochastic neuron, such that for a one dimensional action generation the output (*O_ac_*) is given as:

(13) $$o_{ac}(t)=\epsilon (t)+\sum_{i\;=\;1}^{K}w_{i}(t)u_{i}(t),$$

where *K* denotes the dimension (total number) of sensory stimuli (**u**(*t*)) to the agent being controlled. The parameter *w_i_* denotes the synaptic weights for the different sensory inputs projecting to the actor neuron. Stochastic noise is added to the actor via ϵ(*t*), which is the exploration quantity updated at every time step. This acts as a noise term, such that initially exploration is high, and the agent needs to navigate the environment more if the expected cumulative future reward *v*(*t*) is sub-optimal. However, as the agent learns to successfully predict the maximum cumulative reward (value function) over time, and the net exploration is decreased. As a result ϵ(*t*) gradually tends toward zero as the agent starts to learn the desired behavior (correct policy). Using Gaussian white noise σ (zero mean and standard deviation one) bounded by the minimum and maximum limits of the value function (*v_min_* and *v_max_*), the exploration term is modulated as follows:

(14) $$\epsilon (t)=\Omega \sigma (t)\;\cdot \text{ min}\left[0.5,\text{ max}\left(0,\frac{v_{max}-\hat{v}(t)}{v_{max}-v_{min}}\right)\right].$$

Here, Ω is a constant scale factor selected empirically (see Supplementary Material for details). The actor learns to produce the correct policy, by an online adaptation (Figure 3 left above) of its synaptic weights *w_i_* at each time step as follows:

(15) $$\Delta w_{i}(t)=\tau_{a}\delta (t)u_{i}(t)\epsilon (t),$$

where τ*_a_* is the learning rate such that 0 < τ_*a*_ < 1. Instead of using direct reward *r*(*t*) to update the input to actor neuron synaptic weights, using the TD-error (i.e., error of an internal reward) allows the agent to learn successful behavior, even in cases of delayed reward scenarios (reward is not given uniformly for each time step but is delivered as a constant value after a set of actions were performed to reach a specific goal). In general, once the agent learns the correct behavior, the exploration term (ϵ(*t*)) should become zero, as a result of which no further weight change (Equation 15) occurs and *o_ac_*(*t*) represents the desired action policy, without any additional noise component.

## 3. Results

In order to test the performance of our bio-inspired adaptive combinatorial learning mechanism, and validate the interaction through sensory feedback, between reward-based learning (basal ganglia) and correlation-based learning (cerebellum) systems, we employ a simulated, goal-directed decision making scenario of foraging behavior. This is carried out within a simplified paradigm of a four-wheeled robot navigating an enclosed environment, with gradually increasing task complexity.

### 3.1. Robot model

**Figure 5:**
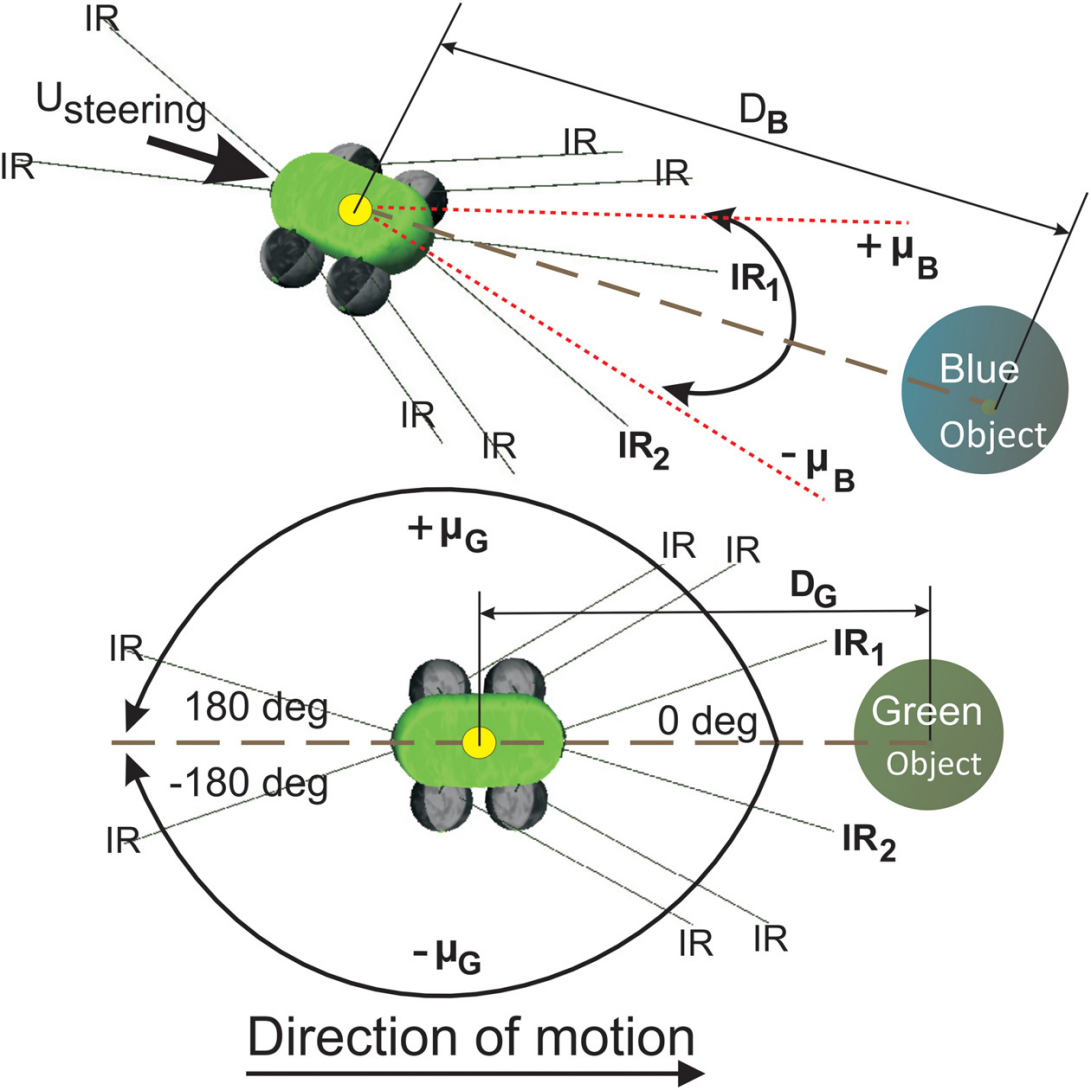
**Simulated mobile robot system for goal-directed behavior task**. **(Top)** The mobile robot NIMM4 with different types of sensors. The relative orientation sensor μ is used as state information for the robot. **(Bottom)** Variation of the relative orientation μ*_G_* to the green goal. the front left and right infrared sensors *IR*_1_ and *IR*_2_ are used to detect obstacles in front of the robot. Direction control for the robot is maintained using the quantity *U_steering_* calculated by the individual learning components (ICO and actor-critic) and then fed to the robot wheels to generate forward motion or steering behavior. Sensors *D_G_* and *D_B_* measure straight line distance to the goal locations.

The simulated wheeled robot NIMM4 (Figure 5) consists of a simple body design with four wheels whose collective degree of rotation controls the steering and the over all direction of motion. It is provided with two front infrared sensors (*IR*_1_ and *IR*_2_) which can be used to detect obstacles to its left or right side, respectively. Two relative orientation sensors (μ_*G*_ and μ*_B_*) are also provided, which can continuously measure the angle of deviation of the robot with respect to the green (positive) and blue (negative) food sources. They are calibrated to take values in the interval [−180°, 180°] with the angle of deviation μ*_G,B_* = 0*^o^* when the respective goal is directly in front of the robot, μ*_G,B_* is positive when the goal locations are to the right of the robot and negative for the opposite case. In addition NIMM4 also consists of two relative position sensors (*D_G,B_*) that can calculate it's relative straight line distance to a goal, taking values in the interval [0, 1], with the respective sensor reading tending to zero, as the robot gets closer to the goal location and vice versa.

### 3.2. Experimental setup

**Figure 6:**
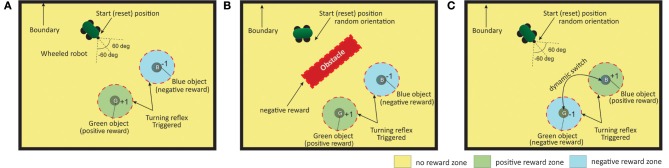
**Three different scenarios for the goal-directed foraging task**. **(A)** Environmental setup without an obstacle case. Green and Blue objects represent the two food sources with positive and negative rewards, respectively. The red dotted circle indicates the region where the turning reflex response (from the ICO learner) kicks in. The robot is started from and reset to the same position, with random orientation at the beginning of each trial episode. **(B)** Environmental setup with an obstacle. In addition to the previous setup, a large obstacle is place in the middle of the environment. The robot needs to learn to successfully avoid it and reach the rewarding food source. Collisions with the obstacle (triggered by *IR*_1_ and *IR*_2_) generate negative rewards (−1 signal) to the robot. **(C)** Environmental setup with dynamic switching of the two objects. It is an extended version of the first scenario. After every 50 trials the reward zones are switched such that the robot has to dynamically adjust to the new positively reinforced location (food) and learn a new trajectory from the starting location.

The experimental setup (Figure 6) consists of a bounded environment with two different food sources (desired vs punishing) located at fixed positions. The primary task of the robot is to navigate the environment such that, eventually, it should learn to steer toward the food source that leads to positive reinforcements (green spherical ball in Figures 6A–C) while avoiding the goal location that provides negative reinforcements or punishments (blue spherical ball), within a specific time interval. The main task is designed as a continuous state-action problem with a distal reward setup (Reinforcement zone in Figure 6), such that the robot starts at a fixed spatial location with random initial orientation ([−60°, 60°]) and receives the positive or negative reinforcement signal only within a radius of specific distance (*D_G,B_* = 0.2) from the two goal locations. Within this boundary, for the green goal it receives a continuous reward of +1 at every time step and a continuous punishment of −1 in case of the blue goal, respectively. At other locations along the environment no reinforcement signal is given to the robot.

The experiments are further divided into three different scenarios of, foraging without an obstacle (case I), with single obstacle (case II) and a dynamic foraging scenario (case III), demonstrating different degrees of reward modulated adaptation between the two learning systems in different environments. In all scenarios, the robot can continuously sense its angle of deviation to the two goals with μ*_G,B_* always active. This acts as a Markov decision process (MDP) such that, the next sensory state of the robot depends on the sensory information for the current state of the robot and the action it performed, and is conditionally independent of all the previous sensory states and actions. Detecting the obstacle results in negative reinforcement (continuous −1 signal) triggered by the front infrared sensors (*IR*_1,2_ > 1.0). Furthermore, hitting the boundary wall in the arena results in a negative reinforcement signal (−1), with the robot being reset to the original starting location. Although the robot is provided with relative distance sensors, sensory stimuli (state information) is provided using only the angle of deviation sensors and the infrared sensors. The reinforcement zone (distance of *D_G,B_* = 0.2) is also used as the zone of reflex to trigger a reflex signal for the ICO learner. Fifty runs were carried out for each setup in all cases. Each run consisted of a maximum of 150 trials. The robot was reset if the maximum simulation time of 15 s was reached, or if it reaches one of the goal locations or if it hits a boundary wall, which ever occurs earlier.

### 3.3. Cerebellar system: ICO learning setup

The cerebellar system in the form of ICO learning (Figure 3 right) was setup as follows: μ*_G,B_* were used as predictive signals (CS). Two independent reflex signals (*x*_0,*B*_ and *x*_0,*G*_, see equation 6) were configured with one for blue food source and the other for the green food source (US). The setup was designed following the principles of delayed conditioning experiments, where, an overlap between the CS and the US stimuli needs to exist in order for the learning to take place. The reflex signal was designed (measured in terms of the relative orientation sensors of the robot) to elicit a turn toward a specific goal once the robot comes within the reflex zone (inside the dotted circle in Figures 6B,C). Irrespective of the kind of goal (desired or undesired) the reflex signal drives the robot toward it with a turn proportional to the deviations defined by μ*_G,B_* i.e., large deviations cause sharper turns. The green and the blue ball were placed such that there was no overlap between the reflex areas, hence only one reflex signal per goal, got triggered at a time. In other words, the goal of the ICO learner is simply to learn to steer toward a food location without any knowledge of it's worth. This is representative of an adaptive reflexive behavior as observed in rodent foraging studies where in the behavior is guided without explicit rewards, but just driven by conditioning between the CS-US stimuli, such that the robot or animal learns to favor certain spots in the environments without any knowledge of their worth. The weights of the ICO learner ρ_μ_*G*__ and ρ_μ_*B*__ (Equation 6) with respect to the green and blue goals were initialized to 0.0. If the positive derivative of the reflex signal becomes greater than a predefined threshold, the weights change and otherwise they remain static, i.e., a higher change in ρ_μ_*G*__ in comparison to ρ_μ_*B*__ would mean that the robot gets drawn toward the green goal more.

### 3.4. Basal ganglia system: reservoir actor-critic setup

The basal ganglia system in the form of a reservoir based actor-critic learner was setup such that, the inputs to the critic and actor networks (Figure 3 left) consisted of the two relative orientation sensor data μ*_G_* and μ_*B*_ and the front left and right infrared sensors (*IR*_1_ and *IR*_2_) of the robot (Figure 4). Although the robot also contains relative distance sensors, these were not used as state information inputs. This makes the task less trivial, such that sufficient but not complete information was provided to the actor-critic RL network. The reservoir network for the critic consisted of *N* = 100 neurons and one output neuron that estimates the value function *v*(*t*) (Equation 10). Reservoir input weights *W_in_* were drawn from an uniform distribution [−0.5, 0.5] while the reservoir recurrent weights *W_sys_* were drawn from a Gaussian distribution of mean 0 and standard deviation *g*^2^/*N* (see Equation 9). Here *g* acts as the scaling factor for *W_sys_*, and it was designed such that there is only 10% internal connectivity in *W_sys_* with a scaling factor of 1.2. The reward signal *r*(*t*) (Equation 12) was set to +1 when the robot comes close (reflex/reinforcement zone) to the green ball and to −1 when it comes close to the blue ball. A negative reward of −1 was also given for any collisions with the boundary walls or obstacle. At all other locations within the environment, the robot receives no explicit reward signal. Thus, the setup is designed keeping a delayed reward scenario in mind, such that earlier actions lead to a positive or negative reward, only when the robot enters the respective reinforcement/reflex zone. The synaptic weights of the actor with respect to the two orientation sensors (*w*_μ_*G*__ and *w*_μ_*B*__) were initialized to 0.0, while the weights with respect to the infrared sensors (*w*_*IR*_1__ and *w*_*IR*_2__) were initialized to 0.5 (equation 13). After learning, a high value of *w*_μ_*G*__ and a low value of *w*_μ_*B*__ would drive the robot toward the green goal location and away from the blue goal. The weights of the infrared sensor inputs effectively control the turning behavior of the robot when encountered with an obstacle (higher *w*_*IR*_1__—right turn, higher *w*_*IR*_2__—left turn). The parameters of the adaptive combinatorial network are summarized in the Supplementary Tables 1–3.

### 3.5. Case I: foraging without obstacle

**Figure 7:**
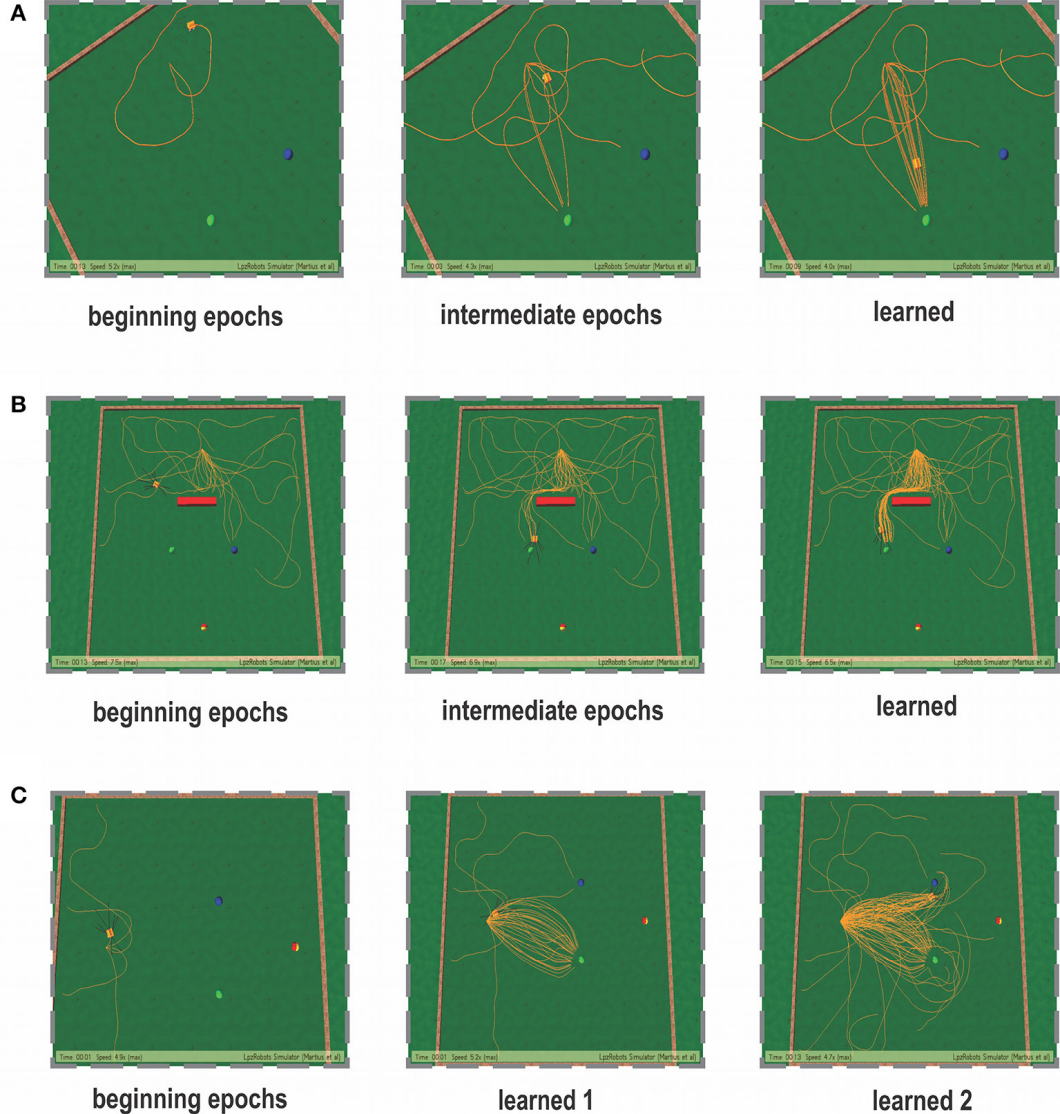
**Simulation snapshots of the robot learning for the three cases taken at specific epochs of time**. **(A)** Snapshots of the learning behavior for the static foraging task without obstacles. **(B)** Snapshots of the learning behavior for the static foraging task with a single obstacle. **(C)** Snapshots of the learning behavior for the dynamic foraging task. Panel learned 1—represents the learned behavior for the initial task of reaching the green goal. After 50 trials, the reward stimulus was changes and the new desired (positively reinforced) location was the blue goal. Panel learned 2—represents the learned behavior after dynamic switching of reward signals.

**Figure 8:**
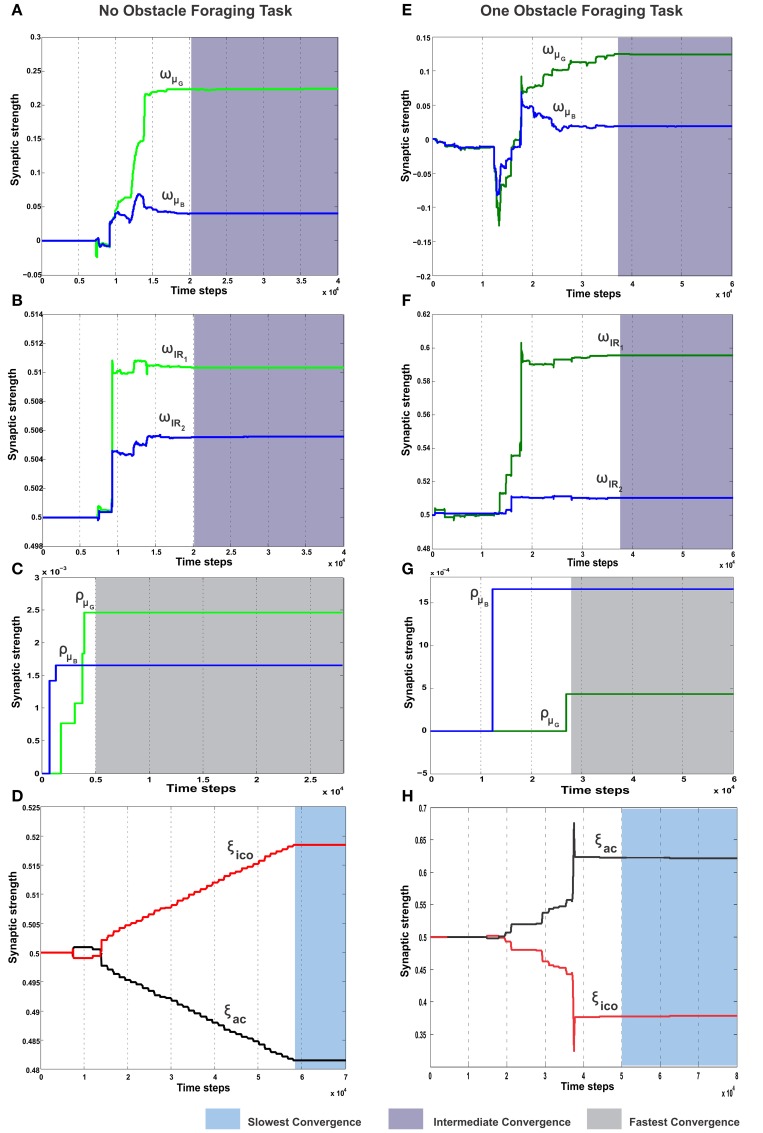
**Synaptic weight change curves for the static foraging tasks without obstacle and with single obstacle**. **(A)** Change in the synaptic weights for actor-critic RL learner. Here *w*_μ_*G*__ corresponds to the input weights of the orientation sensor toward the green goal and *w*_μ_*B*__ corresponds to the input weights of the orientation sensor toward the blue goal. **(B)** Change in the weights of the two infrared sensor inputs of the actor. *w*_*IR*_1__ is the left IR sensor weight, *w*_*IR*_2__ is the right IR sensor weights. **(C)** Change in the synaptic weights of the ICO learner. ρ_μ_*G*__ is the CS stimulus weight for the orientation sensor toward green, ρ_μ_*B*__ the CS stimulus weight for the orientation sensor toward blue. **(D)** Learning curve of the RMHP combined learning mechanism showing the change in the weights of the ICO network output (depicted in red). ξ*_ico_* is weight of the ICO network output. ξ*_ac_* is weight of the actor-critic RL network output (depicted in black). **(E–H)** Show the change in the weights corresponding to the single obstacle static foraging task. In all the plots the gray shaded region marks the region of convergence for the respective synaptic weights. Three different timescales exist in the system, with the ICO learning being the fastest, actor-critic RL being intermediate and the adaptive combined learning being the slowest. (see text for more details.)

**Figure 9:**
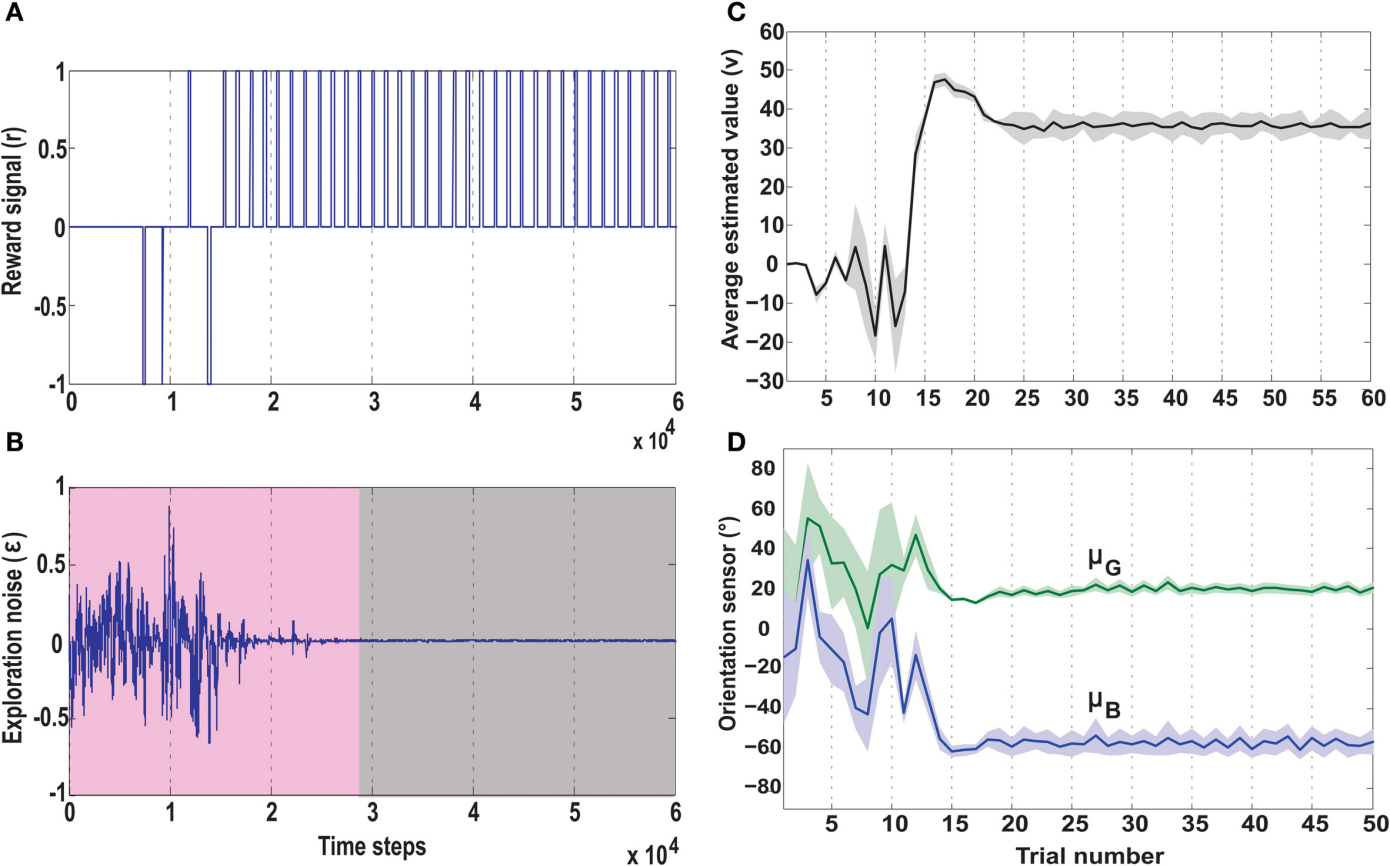
**Temporal development of key parameters of the actor-critic RL network, in the no obstacle foraging task**. **(A)** Development of the reward signal (r) over time. Initially the robot receives a mix of positive and negative rewards due to random explorations. Upon successfully learning the task, the robot is steered toward the green goal every time, receiving only positive rewards. **(B)** Development of the exploration noise (ϵ) for the actor. During learning there is a high noise in the system (pink shaded region), which causes the the synaptic weights of the actor to change continuously. Once the robot starts reaching the green goal more often the TD error from the critic decreases leading to a decrease in exploration noise (gray shaded region), which in turn causes the weights to stabilize (Figure 7). **(C)** Average estimated value (v) as predicted by the reservoir critic is plotted for each trial. The maximum estimated value is reached after about 18 trials after which the exploration steadily decreases and the value function prediction also reaches near convergence at 25 trials (1 trial approximates 1000 time steps). The thick black line represents the average value calculated over 50 runs of the experiment with standard deviation given by the shaded region. **(D)** Plots of the two orientation sensor readings (in degrees) for the green (μ*_G_*) and the blue (μ_*B*_) goals, averaged over 50 runs. During initial exploration the angle of the deviation of the robot from the two goals changes randomly. However, after convergence of the learning rules, the orientation sensor readings stabilize with small positive angle of deviation toward the green goal and large negative deviation from the blue goal. This shows that post learning, the robot steers more toward the green goal and away from the blue goal. Here the thick lines represent average values and the shaded regions represent standard deviation.

In the simplest foraging scenario the robot was placed in an environment with two possible food sources (green and blue) and without any obstacle in between (Figure 6A). In this case the green food source provided positive reward while the blue food source provided negative reward. The goal of the combined learning mechanism was to make the robot successfully steer toward the desired food source. Figure 7A shows simulation snapshots of the behavior of the robot as it explores the environment. As observed from the trajectory of the robot, initially it performed a lot of exploratory behavior and randomly moved around in the environment, but eventually it learned to move solely toward the green goal. This can be further analyzed looking at the development of the synaptic weights of the different learning components as depicted in Figure 8. As observed in Figure 8C due to the simple correlation mechanism of the ICO learner (cerebellar system), the ICO weights adapt relatively faster as compared to the actor. Due to random explorations (Figure 9B) in the beginning, in the event of the blue goal being visited more frequently, reflexive pull toward blue goal - ρ_μ_*B*__ is greater than toward the green goal - ρ_μ_*G*__. However, after sufficient explorations, as the robot starts reaching the green goal more frequently, ρ_μ_*G*__ also starts developing. This is counteracted by the actor weights (basal ganglia system), where in, there is a higher increase in *w*_μ_*G*__ (orientation sensor input representing angle of deviation from green goal) as compared to *w*_μ_*B*__ (orientation sensor input representing angle of deviation from blue goal). This is caused as result of the increased positive rewards received from the green goal (Figure 9A) that causes the TD-error to modulate the actor weights (equation 15) accordingly. At the same time no significant change is seen in the infrared sensor input weights (Figure 8B), due to the fact that in this scenario, the infrared sensors get triggered only on collisions with the boundary wall and remain dormant otherwise. Recall that the infrared sensor weights were initialized to 0.5.

Over time as the robot moves more toward the desired food source, the ICO weights also stabilize with the reflex toward the green goal being much stronger. This also leads to a reduction of the exploration noise (Figure 9B), and the actor weights eventually converge to a stable value (Figures 8A,B). Here, the slow RMHP rule performs a balancing act between the two learning systems with initial higher weight of the actor-critic learner and then a switch toward the ICO system, once the individual learning rules have converged. Figure 9C shows the development of the value function (*v*(*t*)) at each trial, as estimated by the critic. As observed initially the critic underestimates the total value due to high explorations and random navigation in the environment. However, as the different learning rules converge, the value function starts to reflect the total accumulated reward with stabilization after 25 trials (each trials consisted of approximately 1000 time steps).

This is also clearly observed from the change of the orientation sensor readings shown in Figure 9D. Although there is considerable change in the sensor readings initially, after learning, the orientation sensor toward the green goal (μ*_G_*) records positive angle, while the orientation from the blue goal μ_*B*_ records considerably lower negative angles. This indicates that the robot learns to move stably toward the positively rewarded food source and away from the oppositely rewarded blue food source. Although this is the simplest foraging scenario, the development of the RMHP weights ξ*_ico_* and ξ*_ac_* (Figure 8D) depicts the adaptive combination of the basal gangliar and cerebellar learning systems for goal-directed behavior control. Here the cerebellar system (namely ICO) acts as a fast adaptive reflex learner that guides and shapes the behavior of the reward-based learning system. Although both the individual systems eventually converge to provide the correct weights toward the green goal, the higher strength of the ICO component (ξ*_ico_*) leads to a good trajectory irrespective of the starting orientation of the robot. This is further illustrated in the simulation video showing three different scenarios of only ICO, only actor-critic and the combined learning cases, see Supplementary Movie 1.

### 3.6. Case II: foraging with single obstacle

In order to evaluate the efficacy of the two learning systems and their cooperative behavior, the robot was now placed in a slightly modified environment (Figure 6B). As in the previous case, the robot still starts from a fixed location with initial random orientations. However, it now has to overcome an obstacle placed directly in front (field of view), in order to reach the rewarding food source (green goal). Collisions with the obstacle, during learning, resulted in negative rewards (−1) triggered by the front left (*IR*_1_) and right (*IR*_2_) infrared sensors. This influenced the actor-critic learner to modulate the actor weights via TD-error and generate turning behavior around the obstacles. In parallel, the ICO system, still learns only a default reflexive behavior of getting attracted toward either of the food sources by a magnitude proportional to its proximity to them (same as case I), irrespective of the associated rewards. As observed from the simulation snapshots in Figure 7B, after initial random exploration, the robot learns the correct trajectory to navigate around the obstacle and reach the green goal. From the synaptic weight development curves for the actor neuron (Figure 8E) it is clearly observed that although initially there is a competition between *w*_μ_*G*__ and *w*_μ_*B*__, after sufficient exploration, as the robot gets more positive rewards by moving to the green food source, the *w*_μ_*G*__ weight becomes larger in magnitude and eventually stabilizes.

Concurrently in Figure 8F, it can be observed that unlike the previous case the left infrared sensor input weight *w*_*IR*_1__ gets considerably higher as compared to *w*_*IR*_2__. This is indicative of the robot learning the correct behavior of turning right in order to avoid the obstacle and reach the green goal. However, interestingly, as opposed to the simple case (no obstacle) the ICO learner tries to pull the robot more toward the blue goal, as seen from the weight development of ρ_μ_*G*__ and ρ_μ_*B*__ in Figure 8G. This behavior can be attributed to the fact that, as the robot reaches the blue object in the beginning, the fast ICO learner provides high weights for a reflexive pull toward the blue as opposed to the green goal. As learning proceeds and the robot learns to move toward the desired location (driven by the actor-critic system), the ρ_μ_*G*__ weight also increases, however it still continues to favor the blue goal. As a result in order to learn the correct behavior the combined learning systems needs to favor the actor-critic mechanism more as compared to the naive reflexives from the ICO. This is clearly observed from the balancing between the two as depicted in the ξ*_ico_* and ξ*_ac_* weights in Figure 8H. Following the stabilization of the individual learning system weights, the combined learner provides much higher weighting of the actor-critic RL system. Thus, in this scenario, due to the added complexity of an obstacle, one sees that the reward modulated plasticity (RMHP rule) learns to balance the two interacting learning systems, such that the robot still performs the correct decisions overtime (see the simulation run from Supplementary Movie 2).

### 3.7. Case III: dynamic foraging (reversal learning)

**Figure 10:**
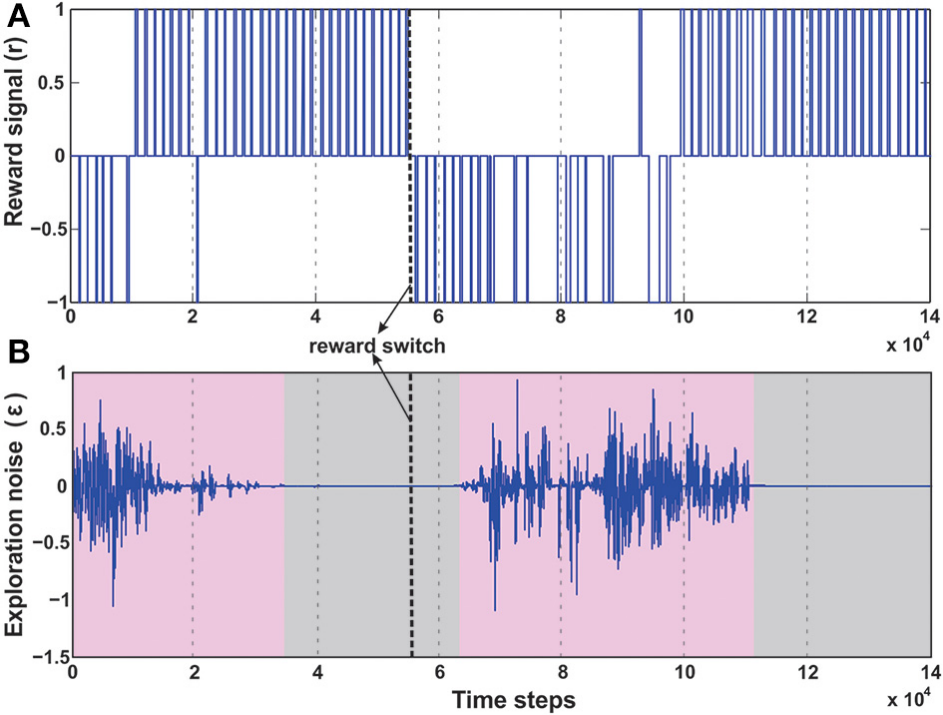
**Temporal development of the reward and exploration noise for the dynamic foraging task**. **(A)** Change in the reward signal (r) over time. Between 3 × 10^4^ time steps and 5 × 10^4^ time steps the robot learns the initial task of reaching the green goal, receiving positive rewards (+1), successively. However, after 50 trials (approximately 5 × 10^4^ to 5.5 × 10^4^ time steps) the reward signals were changed, causing the robot to receive negative rewards (−1) as it drives to the green goal. After around 10 × 10^4^ time steps as the robot learns to steer correctly toward the new desired location (blue goal), it successively receives positive rewards. **(B)** Change in the exploration noise (ϵ) over time. There is random exploration in the beginning of the task and after switching the reward signals (pink shaded regions), followed by stabilization and decrease in exploratory noise once the robot learns the correct behavior (gray shaded region). In both plots the thick dashed line (black) marks the point of reward switch.

A number of modeling as well as experimental studies of decision making (Sugrue et al., 2004) have considered the behavioral effects of associative learning mechanisms on dynamic foraging tasks as compared to static ones. Thus, in order to test the robustness of our learning model, we changed the original setup (Figure 6C), such that, initially a positive reward (+1) is given for the green object and a negative reward (−1) for the blue one. This enables the robot to learn moving toward the green object while avoiding the blue object. However, after every 50 trials the sign of the rewards was switched such that now the blue object received positive reward, and the green goal the opposite. As a result the learning system needs to quickly adapt to the new situation and learn to navigate to the correct target. As observed in the Figure 10B initially the robot performs random explorations receiving a mixture of positive and negative rewards, however after sufficient trials, the robot reaches a stable configuration (exploration drops to zero) and receives positive rewards concurrently (Figure 10A). This corresponds to the previous case of learning to move toward the green goal. As the rewards were switched, the robot then obtained negative reward when it moved to the green object. As a consequence, the exploration gradually increased again; thereby the robot also exhibited random movements. After successive trials, a new stable configuration was reached with the exploration dropping to zero and now the robot received more positive rewards, however for the other target (blue object). This is depicted with more clarity, in the simulation snapshots in Figure 7C (beginning—random explorations, learn 1—reaching green goal, learn 2—reaching blue goal).

**Figure 11:**
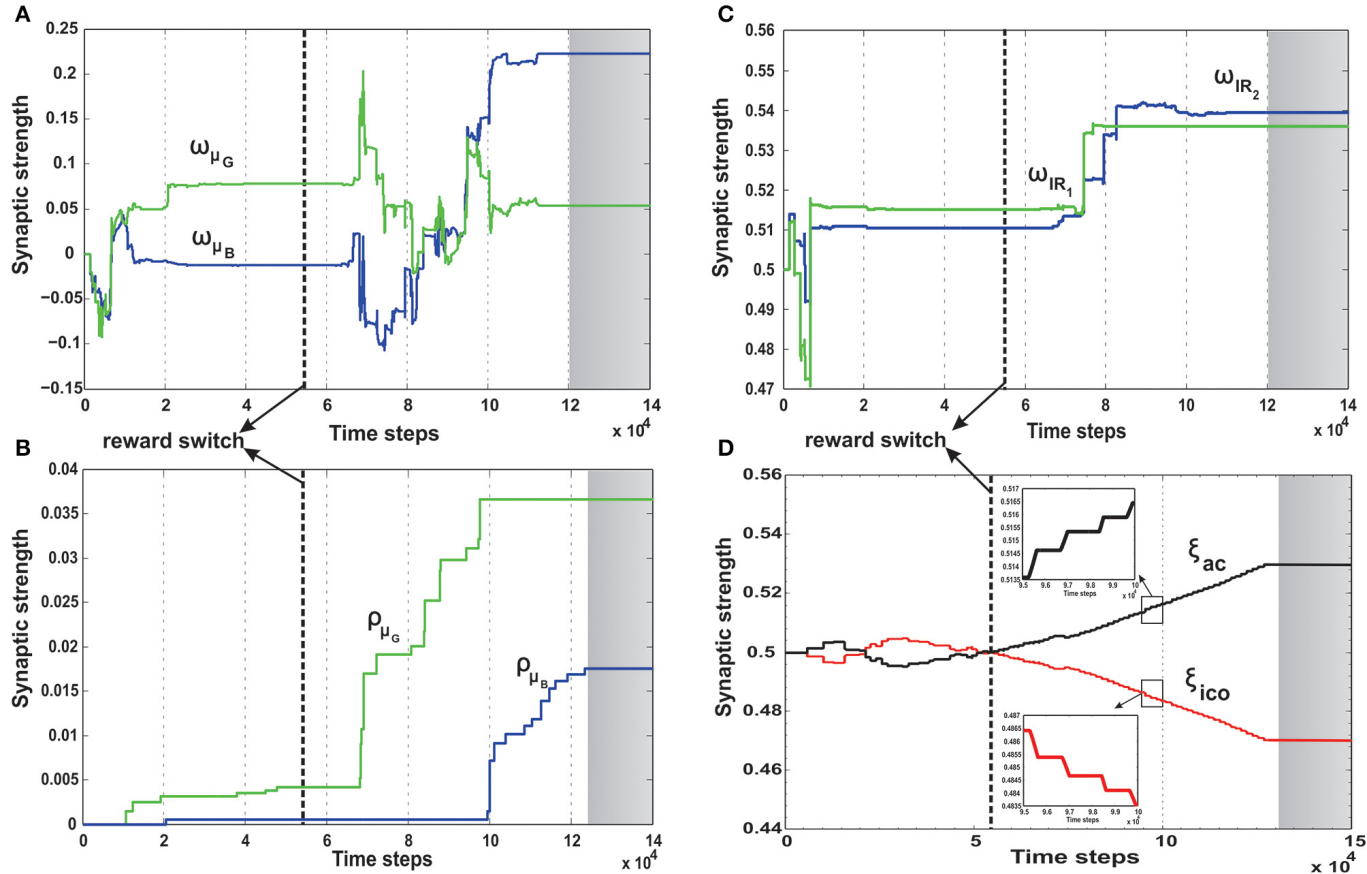
**Synaptic weight change curves for the dynamic foraging task**. **(A)** Change in the synaptic weights for actor-critic RL learner. Here *w*_μ_*G*__ corresponds to the input weights of the orientation sensor toward the green food source (spherical object) and *w*_μ_*B*__ corresponds to the input weights of the orientation sensor toward the blue. **(B)** Change in the synaptic weights of the ICO learner. ρ_μ_*G*__—the CS stimulus weight for the orientation sensor toward green, ρ_μ_*B*__ the CS stimulus weight for the orientation sensor toward blue. **(C)** Change in the weights of the two infrared sensor inputs to the actor. *w*_*IR*_1__—left IR sensor weight, *w*_*IR*_2__—right IR sensor weights. Modulation of the IR sensor weights initially and during the periods 7 × 10^4^ - 9 × 10^4^ time steps can be attributed to the high degree of exploration during this time, where in the robot has considerable collisions with the boundary walls triggering these sensors (see Figure 7C). **(D)** Learning curve of the RMHP combined learning mechanism showing the change in the weights of the individual components. ξ*_ico_*—weight of the ICO network output (depicted in red), ξ*_ac_*—weight of the actor-critic RL network output (depicted in black). Here the ICO weights converge initially for the first part of the task, however fail to re-adapt upon change of reward signals. This is counter balanced by the correct evolution of the actor weights. As a result although initially the combinatorial learner places higher weight for the ICO network, after task switch, due to change in reinforcements the actor-critic RL system receives higher weights and drives the actual behavior of the robot. The inlaid plots show a magnified view of the two synaptic weights between 9.5 × 10^4^ - 10 × 10^4^. The plots show that the weights do not change in a fixed continuous manner, but increase/decrease in a step like formation corresponding to the specific points of reward activation (Figure 10A). In all the plots the gray shaded region mark the region of convergence for the respective synaptic weights, and the thick dashed line (black) marks the point of reward switch. (see text for more details).

In order to understand how the combined learning mechanism handles this dynamic switching, in Figure 11 we plot the synaptic weight developments of the different components.

Initially the robot behavior is shaped by the ICO weights (Figure 11B) which learn to steer the robot to the desired location, such that the reflex toward green object (ρ_μ_*G*__) is stronger than that toward the blue object (ρ_μ_*B*__). Furthermore, as the robot receives more positive rewards, the basal ganglia system starts influencing it's behavior by steadily increasing the actor weights toward the green object (Figure 11A, *w*_μ_*G*__, *w*_*IR*_1__ > *w*_μ_*B*__, *w*_*IR*_2__). This eventually causes the exploration noise (ϵ) to decrease to zero and the robot learns a stable trajectory toward the desired food source. This corresponds to the initial stable region of the synaptic weights between 2 × 10^4^ and 6 × 10^4^ time steps in Figures 11A–C. Interestingly the adaptive RMHP rule tries to balance the influence from the two learning systems with eventual higher weighting of the ICO learner. This is similar to the behavior observed in the no obstacle static scenario (Figure 8D). After 50 trials (5 × 10^4^ time steps), the reward signs were inverted which causes the exploration noise to increase. As a result the synaptic weights try to adapt once again and influence the behavior of the robot, now toward the blue object. In this scenario although the actor weights eventually converge to the correct configuration of *w*_μ_*B*__ greater than *w*_μ_*G*__, the cerebellar reflexive behavior remains biased toward the green object (previously learned stable trajectory). This can be explained from the fact that the cerebellar or ICO learner has no knowledge of the type of reinforcement received from the food sources, and just naively tries to attract the robot to a goal when it is close enough (within the zone of reflex) to it. As a result of this behavior, the RMHP rule tries to balance the contributions of both learning mechanisms (Figure 11D), by increasing the strength of the actor-critic RL component as compared to the ICO learner component (ξ_*ac*_ > ξ_*ico*_). This lets the robot, now learn the opposite behavior of stable navigation toward the blue food source, causing the exploration noise to decrease once again. Thus, through the adaptive combination of the different learning systems, modulated by the RMHP mechanism, the robot was able to deal with dynamic changes in environment and complete the foraging task successfully (see the simulation run in Supplementary Movie 3).

**Figure 12:**
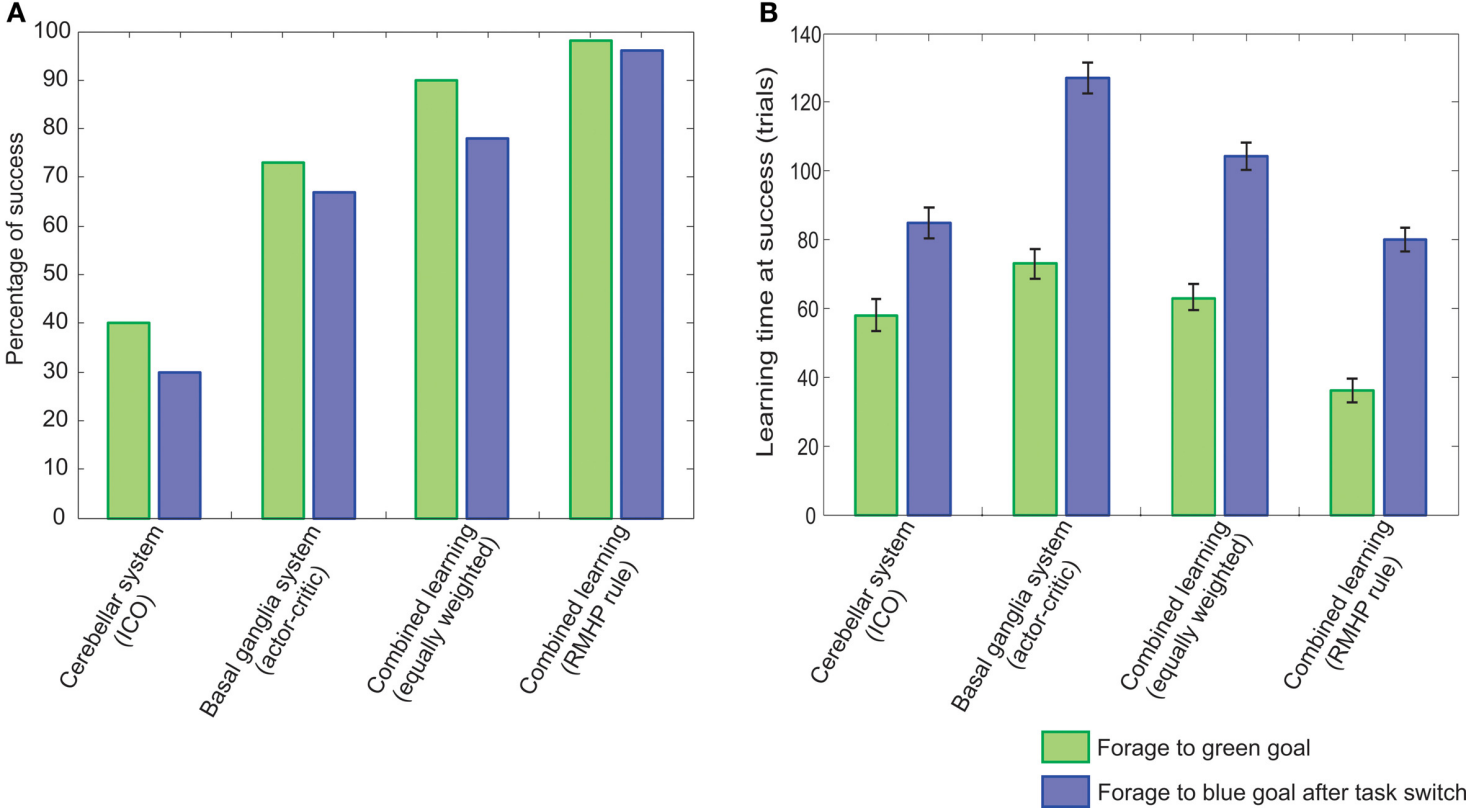
**Comparison of performance of RMHP modulated adaptive comninatorial learning system for the dynamic foraging task**. **(A)** Percentage of success measured over 50 experiments. **(B)** Average learning time (trials needed to successfully complete the task, calculated over 50 experiments (error bars indicate standard deviation with 98% confidence intervals). In both cases the green bars represent the performance for the initial task of learning to reach the green goal, while blue bars represent the performance in the subsequent task after dynamic switching of reward signals.

Furthermore, as observed from the rate of success on the dynamic foraging task (Figure 12A), the RMHP based adaptive combinatorial learning mechanism clearly outperforms the individual systems (only ICO or only actor-critic RL). Here the rate of success was calculated as the percentage of times the robot was able to successfully complete the first task of learning to reach the green food source (green colored bars), and then after switching of the rewards signals, the percentage of times it successfully reached the blue food source (blue colored bars). Furthermore, in order to test the influence of the RMHP rule, we tested the combined learning with both, equal weightage to ICO and actor-critic systems as well as a plasticity induced weighting for the two individual learning components. It was observed that although for the initial static case of learning to reach the green goal the combined learning mechanism with equal weights works well, the performance drops considerably, after the reward signals were switched, and re-adaptation was required. Such a performance was also observed in our previous work (Manoonpong et al., 2013) using a simple combined learning model of feed-forward actor-critic (radial basis function) and ICO learning. However, in this work we show that the combination of a recurrent neural network actor-critic with ICO learning, using the RMHP rule, was able to re-adapt the synaptic weights and combine the two systems effectively. The learned behavior greatly outperforms the previous case and shows a high success rate for both, the initial navigation to green goal location and successively to the blue goal location, after switching of reinforcement signals.

In Figure 12B, we plot the average time taken to learn the first and second part of the dynamic foraging task. The learning time was calculated as the number of trials required on successful completion of the task (i.e., successively reaching green or blue goal/food source location) averaged over 50 runs of the experiment. The combined learning mechanism with RMHP, successfully learns the task in less trials, as compared to the individual learning systems. However there was a significant increase in the learning time after the switching of reward signals. This can be attributed to the fact that after exploration goes to zero initially, a stable configuration is reached, the robot needs to perform more random explorations in order to change the strength of the synaptic connections considerably such that the opposite action of steering to the blue goal can be learned. Furthermore, as expected from the relatively fast learning rate of the ICO system, it was able to learn the tasks much quicker as compared to the actor-critic system, however its individual performance was less reliable than the actor-critic system as observed from the success rate (Figure 12A). Taken together, our model of RMHP induced combination mechanism provides a much more stable and fast decision making system as compared to the individual systems or a simple naive parallel combination of the two.

## 4. Discussion

Numerous animal behavioral studies (Lovibond, 1983; Brembs and Heisenberg, 2000; Barnard, 2004) have pointed to an interactive role of classical and operant conditioning in guiding the decision making process for goal-directed learning. Typically a number of these psychology experiments reveal compelling evidence that both birds and mammals, can effectively learn to perform sophisticated tasks when trained using a combination of these mechanisms (Staddon, 1983; Shettleworth, 2009; Pierce and Cheney, 2013). The feeding behavior of Aplysia have also been used as model systems in order to compare classical and operant conditioning at the cellular level (Brembs et al., 2004; Baxter and Byrne, 2006) and also study how predictive memory can be acquired by the neuronal correlates of the two learning paradigms (Brembs et al., 2002).

In case of the mamalian brain recent experimental evidence (Neychev et al., 2008; Bostan et al., 2010) point toward the existence of direct communication and interactive combination between the neural substrates of reward learning and delay conditioning learning systems, namely the basal ganglia and the cerebellum. However, the exact mechanism by which these two neural systems interact is still largely unknown. Few experimental studies suggest that such a communication could exists via the thalamus (Sakai et al., 2000), through which reciprocal connections from these two areas connect with the cortical areas in the brain (see Figure 1) (McFarland and Haber, 2002; Akkal et al., 2007). As such, in this paper we make the hypothesis (neural combined learning) that such a combination is driven by a reward modulated heterosynaptic plasticity (Legenstein et al., 2008; Hoerzer et al., 2012), triggered by dopaminergic projections (García-Cabezas et al., 2007; Varela, 2014) existing at the thalamus that dynamically combines the output from the two areas and drives the overall goal directed behavior of an organism. It is important to note that, it is also possible that thalamic projections carrying basal-ganglia and cerebellar inputs could eventually converge onto a single pyramidal cell via relay neurons at the motor cortex. Furthermore, as the motor and frontal cortical regions together with the striatum, have been observed to receive particularly dense dopaminergic projections from the mid brain areas (VTA) (Hosp et al., 2011), it is plausible that the proposed neuromodulatory heterosynaptic plasticity could also occur directly at the cortex (Ni et al., 2014). We model the classical delay conditioning paradigm observed in the cerebellum with the help of input correlation learning (Porr and Wörgötter, 2006), while reward based learning modulated by prediction errors, is modeled using a temporal difference model of actor-critic learning. Using a simple robot model, and three different scenarios of increasing complexity for a foraging task, we demonstrate that the neural combinatorial learning mechanism can effectively and robustly enable the robot to move toward a desired food source while learning to avoid a negatively rewarded, undesired food source while being considerably robust to dynamic changes in the environmental setup.

Although there have been a few robot studies, trying to model basal ganglia behavior (Gurney et al., 2004; Prescott et al., 2006) and cerebellar learning for classical conditioning (Verschure and Mintz, 2001; Hofstoetter et al., 2002), to the best of our knowledge they have only been applied individually. In this study, for the first time, we show how such a combined mechanism can be implemented using a wheeled robot that leads to a more efficient decision making strategy. Although designed with a simplified level of biological abstraction, our model sheds light toward the way basal gangliar and cerebellar structures in the brain indirectly interact with each other through sensory feedback. Furthermore, our model of the critic based on a reservoir network, takes into account the strong reciprocal recurrent connections in the cortex that provide input to the striatal system (this is analogous to the output layer in our model) while being modulated by dopaminergic neural activity (TD-error). Such reservoir models of the basal ganglia system have also been previously implemented in the context of learning language accusation (Hinaut and Dominey, 2013) or for modeling the experimentally observed varying timescales of neural activity of domapinergic neurons (Bernacchia et al., 2011). Specifically in this work, the reservoir also provides a fading memory of incoming sensory stimuli (Dasgupta et al., 2014) that can enable the robot to deal with partially observable state space problems as shown previously in Dasgupta et al. (2013b). As a result such a recurrently connected network typically outperforms non-linear feed-forward models of the critic (Morimoto and Doya, 1998). Although beyond the scope of the current article, our work with the reservoir based critic sheds new insights in to how large recurrent networks can be trained in a non-supervised manner using reward modulation and a simple recursive least squares algorithm, which has hitherto been a difficult problem, with only few simple models existing that work on synthetic data (Hoerzer et al., 2012) or require supervised components (Koprinkova-Hristova et al., 2010).

In the context of goal directed behavior, one may also draw similarity of the basic reflexive mechanism learned by the cerebellum (Yeo and Hesslow, 1998) to innate or intrinsic motivations in biological organisms, in contrast to more extrinsic motivations (in the form of reinforcing evaluative feedbacks) provided by the striatal dopaminergic system of the basal ganglia (Boedecker et al., 2013). Our hypothesis is that in order for an organism to make decisions in a dynamic environment, where in, certain behaviors result in basic reflexes (based on CS—US conditioning) while others lead to specific rewards or punishments, it needs a mechanism that can effectively combine these, in order to accomplish the desired goal. Our neuromodulation scheme, namely, the RMHP rule provides such an adaptive combination that guides the behavior of the robot over time in order to achieve stable goal directed objectives. Particularly, our RMHP based combined learning model provides evidence that cooperation between reinforcement learning and correlation learning systems can enable agents to perform fast and stable reversal learning (adaptation to dynamic changes in the environment). Such combination mechanisms could be crucial in dealing with navigation scenarios involving contrasting or competing goals, with gradual or sudden changes to environmental conditions. Furthermore, this could also point toward possible adaptation or mal-adaptation between the basal ganglia and cerebellum in case of neurological movement disorders like dystonia (Neychev et al., 2008) which typically involve both these brain structures.

Over all our computational model based on the combinatorial learning hypothesis shows that indeed the learning systems of the basal ganglia and the cerebellum can adaptively balance the output of each other in order to deal with changes in environment, reward conditions, and dynamic modulation of pre-learned decisions. Although here we modeled a novel reward modulation between the two systems, no direct feedback (interaction) between the cerebellum and basal ganglia was provided. In the future we plan to include such direct communication between the two in the form of inhibitory feedback, as evident from recent experimental studies (Bostan et al., 2010). However, in its current form, we envision such an adaptive combinatorial learning approach to have wide impact on bio-mimetic agents, in order to provide better solutions of decision making problems in both static and dynamic situations, as well as show how the neuromodulation of executive circuits in the brain can effectively balance output from different areas. While our combined learning model verifies that the adaptive combination of the learning systems of the basal ganglia and the cerebellum leads to effective goal-directed behavior control in an artificial system, it would be interesting to further investigate this combination in biological systems, particularly in terms of the underlying neuronal correlates. As observed by Williams and Williams (1969) in a pigeon pecking at an illuminated key in a Skinner box, their results suggest that the desired key-pecking behavior CR may be shaped (autoshaping) by not only operant conditioning but also by classical conditioning; since imposing an omission schedule on the key-light, key-peck association did little to revoke the conditional pecking response. Hence, it seems that the existing occasional pairing of the key-light CS with the food US are adequate to drive the pecking behavior (CR), which thus emerge from classical conditioning. Based on these principles, several animal behavioral studies have observed similar autoshaping effects even in rodents (Cleland and Davey, 1983; Meyer et al., 2014), where, multiple sources of information (e.g., colored lights or sound (conditioned stimuli), food (reward or unconditioned stimuli), and response levers or keys shape and guide the animal responses over time toward desired behaviors. Although both the basal ganglia (Winstanley et al., 2005) and the cerebellum (Klopf, 1988) have been studied with regards to such behaviors, it has been largely carried out separately. However, our results on artificial systems indicate that their combined learning produces more efficient goal directed behaviors, specially in reversal learning (dynamic foraging) scenarios. As such, future neurobiological (combining lesion and tracing studies) and animal psychology experiments could investigate classical conditioning (correlation-based learning) in the cerebellum, operant conditioning (reward-based learning) in the basal ganglia and their combination for goal-directed behavior control in animals like rodents or birds. Furthermore, although we specifically investigated goal-directed behaviors in this study, there is wide spread evidence of habit learning (Yin and Knowlton, 2006) and motor-skill learning (Salmon and Butters, 1995) in both these brain structures and their implications on neurodenerative diseases like parkinson (Redgrave et al., 2010). Future experimental studies based on this combined learning hypothesis could investigate how the such a combination and interaction between the two learning systems influence goal directed decisions making vs habitual behaviors and the effect on neurodegenrative diseases by possible imbalances between them (de Wit et al., 2011).

## Author contributions

Conceived and designed the experiments: Sakyasingha Dasgupta, Poramate Manoonpong, and Florentin Wörgötter. Performed the experiments: Sakyasingha Dasgupta. Analyzed the data: Sakyasingha Dasgupta and Poramate Manoonpong. Wrote the paper: Sakyasingha Dasgupta. Read and commented on the paper: Poramate Manoonpong and Florentin Wörgötter.

### Conflict of interest statement

The authors declare that the research was conducted in the absence of any commercial or financial relationships that could be construed as a potential conflict of interest.

## Footnotes

^1^Agent here refers to any artificial or biological organism situated in a given environment.

^2^It is also plausible that integration of activity arising in basal ganglia and cerebellum might take place in the thalamus nuclei other than the VL-VA, since pallidal as well as cerebellar fibers are known histologically to terminate not only in the VL-VA but also in other structures (Mehler, 1971).

^3^The reflex signal is typically a default response to an unwanted situation. This acts as the unconditional stimulus occurring later in time, than the predictive conditional stimulus.

^4^This x(t) is different from the neural state activation vector **x**(*t*) of Equation 9.

^5^In reinforcement learning, policy refers to the set of actions performed by an agent that maximizes it's average future reward.

^6^The discount factor helps assigning decreasing value to rewards further away in the past as compared to the current reward.
