# Supplementary material for: Neuromodulatory adaptive combination of correlation-based learning in cerebellum and reward-based learning in basal ganglia for goal-directed behavior control
Source: Front Neural Circuits. 2014 Oct 28;8:126. doi: 10.3389/fncir.2014.00126 (PMC4211401; doi:10.3389/fncir.2014.00126)
Supplement: Supplementary file 4 [file DataSheet1.PDF]

Frontiers in Neural Circuits

Research Article
29 August 2014

Supplementary Material: Neuromodulatory Adaptive Combination of Correlation-based Learning in Cerebellum and Reward-based Learning in Basal Ganglia for Goal-directed Behavior Control

Sakyasingha Dasgupta [1,2*], Florentin Wörgötter [1,2] and Poramate Manoonpong [2,3]

[1] *Institute for Physics - Biophysics, George-August-University, Göttingen, Germany*
[2] *Bernstein Center for Computational Neuroscience, George-August-University, Göttingen, Germany*
[3] *Center for Biorobotics, Maersk Mc-Kinney Møller Institute, University of Southern Denmark, Odense, Denmark*

Correspondence*:
Sakyasingha Dasgupta
Bernstein Center for Computational Neuroscience, George-August-University, Friedrich-hund platz 1, 37077 Göttingen, Germany, sdasgup@gwdg.de

Neuromodulation of Executive Circuits

1 ADDITIONAL EXPERIMENTAL RESULTS

Dopaminergic neurons are primarily believed to encode a reward prediction error (RPE) signal (**Schultz and Dickinson**, 2000). Although, recent experimental evidences have shown that a subset of the VTA dopaminergic neurons can directly encode the reward signal, most of them still follow the canonical RPE coding (**Cohen et al.**, 2012). In the context of the actor-critic reservoir model of the basal ganglia, the temporal difference error (TD-error) is considered as the prediction error signal output of the dopaminergic neurons (**Suri and Schultz**, 2001). As such, in order to test the stability and efficiency of the reward modulated heterosynaptic (RMHP) combined learning rule while using the TD-error ($\delta(t)$) as the neuromodulatory signal at the motor thalamic junction instead of the instantaneous reward signal $r(t)$, we modified equation 2 and equation 3 as follows:

$$\Delta\xi_{ico}(t) = \eta\delta(t)[o_{ico}(t) - \bar{o}_{ico}(t)]o_{ac}(t), \tag{1}$$
$$\Delta\xi_{ac}(t) = \eta\delta(t)[o_{ac}(t) - \bar{o}_{ac}(t)]o_{ico}(t). \tag{2}$$

Here, the TD-error signal($\delta(t)$) is calculated as part of the reservoir critic network and updated based on the current reward and the estimated sum of future rewards ($\hat{v}(t)$) at every time time step as follows:

$$\delta(t) = r(t) + \gamma\hat{v}(t) - \hat{v}(t-1). \tag{3}$$

We tested the performance of the modified learning rule on the foraging scenario with a single obstacle (main text - Figure 6(B)) with no changes to the experimental setup. 20 runs were carried out with the

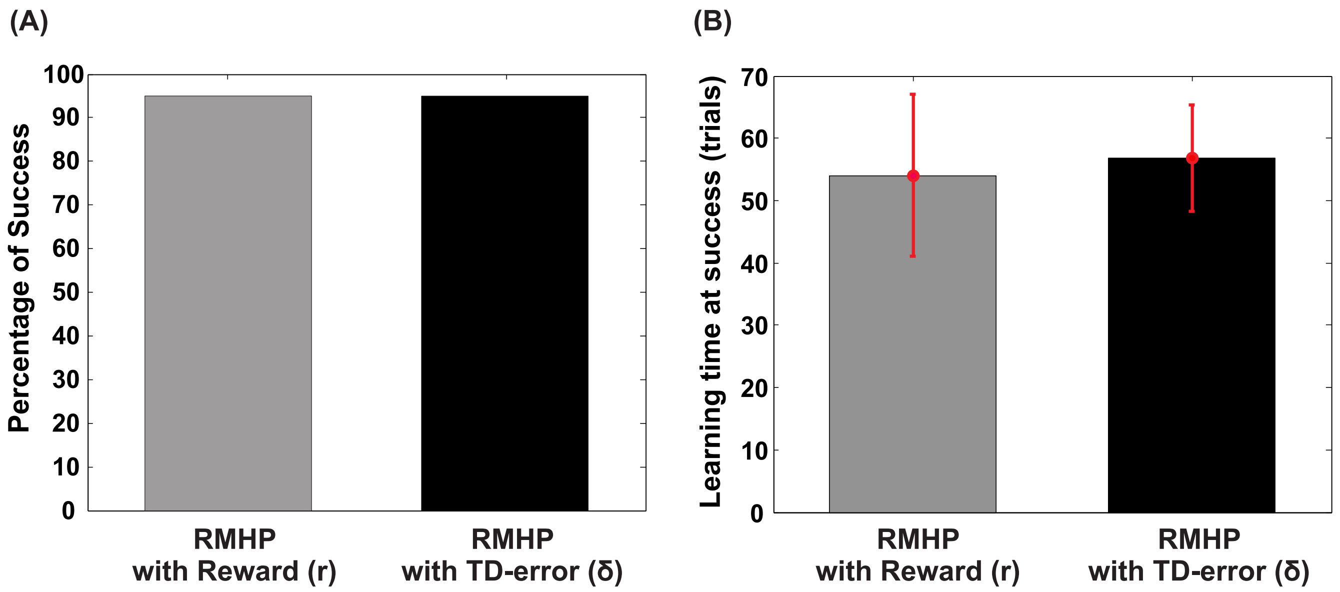

Figure 1. Comparison of performance of RMHP modulated adaptive comninatorial learning system with direct reward (original) and TD-error modulation, in the single obstacle foraging case. **(A)** Percentage of success measured over 20 experiments. **(B)** Average learning time (trials needed to successfully complete the task, calculated over 20 experiments (error bars indicate standard deviation with 98% confidence intervals). In both cases the grey bars represent the performance for task of learning to reach the green goal with the original RMHP rule , while black bars represent the performance in the same task using the TD-error modulated RMHP rule.

original RMHP rule (direct reward signal modulation) and the modified RMPH rule (TD-error modulation). As observed in Figure S1(A), the robot was successfully able to complete the task with only a single failure, achieving a performance rate of 95% in both cases. Figure S1(B) shows the average learning time needed to learn the task under both conditions. The TD-error based learning rule took negligibly longer time to converge to a solution (57 trials) in comparison to the instantaneous reward-based learning rule (54 trials). This behavior can be attributed to the fact that, the TD-error signal is updated continuously resulting in the ICO learner(ξ_{ico}) and the actor-critic learner (ξ_{ac}) weights changing all the time. This is avoided in the direct reward based RMHP rule, since the reward signal $r(t)$ is active only within the positive or negative reward zone and zero otherwise. As a result, any initial wrong estimates by the critic do not effect the combined learning weights, substantially.

Over all our results prove that the RMHP combined learning rule works stably with similar levels of performance, independent of the choice of the instantaneous reward or the temporal difference error as the modulatory signal. However, in this work we have only tested goal-directed decision making scenarios. In other learning scenarios like dynamic motion control (**Barto et al.**, 1983), (**Morimoto and Doya**, 2001) there may be differences in performance for the two variants of the RMHP rule. This would require further analysis under various environmental conditions and goes beyond the scope of the current paper. In essence the current scheme of RMHP (in both variants of direct reward and TD-error modulations) provides an effective and efficient mechanism to combine the reward learning and correlation learning systems of the basal ganglia and the cerebellum brain structures, respectively.

REFERENCES

Barto, A. G., Sutton, R. S., and Anderson, C. W. (1983), Neuronlike adaptive elements that can solve difficult learning control problems, *Systems, Man and Cybernetics, IEEE Transactions on*, , 5, 834–846

Cohen, J. Y., Haesler, S., Vong, L., Lowell, B. B., and Uchida, N. (2012), Neuron-type-specific signals for reward and punishment in the ventral tegmental area, *Nature*, 482, 7383, 85–88

Morimoto, J. and Doya, K. (2001), Acquisition of stand-up behavior by a real robot using hierarchical reinforcement learning, *Robotics and Autonomous Systems*, 36, 1, 37–51

Schultz, W. and Dickinson, A. (2000), Neuronal coding of prediction errors, *Annual review of neuroscience*, 23, 1, 473–500

Suri, R. E. and Schultz, W. (2001), Temporal difference model reproduces anticipatory neural activity, *Neural Computation*, 13, 4, 841–862
