# Supplementary material for: Neuromodulatory adaptive combination of correlation-based learning in cerebellum and reward-based learning in basal ganglia for goal-directed behavior control
Source: Front Neural Circuits. 2014 Oct 28;8:126. doi: 10.3389/fncir.2014.00126 (PMC4211401; doi:10.3389/fncir.2014.00126)
Supplement: Supplementary file 5 [file DataSheet2.PDF]

Appendix Table 1. Parameters of the actor-critic reinforcement learning network

Time constant of the reservoir critic (τ - equation (9))	1s
Reservoir critic size (N - neurons)	100
Forgetting factor (γ - equation (8))	0.98
Critic scaling factor (g - equation (9))	1.2
Critic bias input (b - equation (9))	0.001
Learning rate of critic (λ_{RLS} - algorithm (1))	0.85
Auto-correlation matrix constant (β - algorithm (1))	10^{-2}
Exploration scale factor (Ω - equation (14))	5
Maximum value function (v_{max} - equation (14))	50
Minimum value function (v_{min} - equation (14))	-50
Learning rate of actor (τ_a - equation(15))	0.005
Critic input weights (W_{in})	fixed Uniform [-0.5,0.5]
Critic recurrent weights (W_{sys})	fixed Normal (0, g^2/N)
Recurrent connection probability (W_{sys})	0.1
Critic output weights (W_{out})	plastic
Initialization of actor weights (w_{mu_G} and w_{mu_B})	0.0
Initialization of actor weights (w_{IR_1} and w_{IR_2})	0.5
Number of inputs (K)	4
Number of output	1

Algorithm 2 : Adaptive Neural combinatorial learning algorithm

1: *Input:*

- Actor-critic RL: input stimuli vector $u_{1,2,3,4} = \mu_G, \mu_B, IR_1, IR_2$
- ICO learning: input stimuli vector $x_{1,2} = \mu_G, \mu_B$

2: *Initialization:*

- ICO weights: $\rho_{\mu_G}, \rho_{\mu_B} = 0.0$; $\rho_0 = 1.0$ (reflex signal strength)
- Actor weights: $w_{\mu_G}, w_{\mu_B} = 0.0$; $w_{IR_1}, w_{IR_1} = 0.5$
- RMHP combined learner weights: $\xi_{ico}, \xi_{ac} = 0.5$
- exploration noise ϵ: approximately normal distribution calculated as sum of 'n' i.i.d r.v $\in$ U(0,1)

3: Observe reflex signal x_0 and the sensory signals $x_{1,2}(t)$ and $u_{1,2,3,4}(t)$
4: while (i < max time steps) do
5: Execution:

- $o_{ico}(t) \leftarrow \rho_0 x_0(t) + \sum_{j=1}^{K} \rho_j(t) x_j(t)$
- $o_{ac}(t) \leftarrow \epsilon(t) + \sum_{i=1}^{K} w_i(t) u_i(t)$
- $o_{com}(t) \leftarrow \xi_{ico} o_{ico}(t) + \xi_{ac} o_{ac}(t)$

6: Perform action
7: Observe new sensory states $x'(t)$, $u'(t)$ and new reflex signal $x'_0(t)$
8: Update the reward signal $r(t)$:
if robot is within the green reward zone ($D_G < 0.2$) **then**
$r(t) = +1$
end if
if robot is within the blue reward zone ($D_B < 0.2$) **then**
$r(t) = -1$
end if
if $IR_1 > 1.0$ or $IR_2 > 1.0$ **then**
$r(t) = -1$
end if
9: Update value prediction from critic:

- $\tau \dot{\mathbf{x}}(t) \leftarrow -\mathbf{x}(t) + g \mathbf{W}_{sys} \mathbf{z}(t) + \mathbf{W}_{in} \mathbf{u}(t) + \mathbf{b}$
- $\hat{v}(t) \leftarrow \mathtt{tanh}(\mathbf{W}_{out} \mathbf{z}(t))$

10: Update exploration noise:

- $\epsilon(t) \leftarrow \Omega \sigma(t) \cdot \mathtt{min}\Big[0.5, \mathtt{max}\Big(0, \frac{v_{max} - \hat{v}(t)}{v_{max} - v_{min}}\Big)\Big]$

11: Calculate temporal difference (prediction) error :

- $\delta(t) \leftarrow r(t) + \gamma \hat{v}(t) - \hat{v}(t-1)$.

12: Update all synaptic weights:

- ICO weights : $\frac{d}{dt}\rho_j(t) \leftarrow \mu x_j(t) \frac{d}{dt} x_0(t)$
- Critic weights: $\mathbf{W}_{out}(t) \leftarrow \mathbf{W}_{out}(t-1) + K(t)e(t)$ (see RLS algorithm 1 in main text)
- Actor weights: $\Delta w_i(t) \leftarrow \tau_a \delta(t) u_i(t) \epsilon(t)$
- RMHP weights: $\Delta \xi_{ico}(t) \leftarrow \eta r(t)(o_{ico}(t) - \bar{o}_{ico}(t)) o_{ac}(t)$; $\Delta \xi_{ac}(t) \leftarrow \eta r(t)(o_{ac}(t) - \bar{o}_{ac}(t)) o_{ico}(t)$

Appendix Table 2. Parameters of the input correlation learning (ICO) network

Strength of reflex signal (ρ_0 - equation (6))	1.0
Learning rate (μ - equation(7))	0.001
Initialization of input weights (ρ_{μ_G} and ρ_{mu_B})	0.0
Number of inputs (K)	2
Number of output	1

Appendix Table 3. Parameters of the combinatorial learner (RMHP rule)

Initialization of individual learner weights (ξ_{ico} and ξ_{ac})	0.5
Learning rate (η - equations (2) and (3))	0.0005
